# Association of Optimal Gestational Weight Gain Ranges With Perinatal Outcomes Across Body Mass Index Categories in Twin Pregnancies

**DOI:** 10.1001/jamanetworkopen.2022.22537

**Published:** 2022-07-19

**Authors:** Dongxin Lin, Xuqiong Huang, Dazhi Fan, Gengdong Chen, Pengsheng Li, Jiaming Rao, Huishan Zhang, Xiaoling Guo, Caihong Luo, Zhengping Liu

**Affiliations:** 1 Foshan Institute of Fetal Medicine, Southern Medical University Affiliated Maternal and Child Health Hospital of Foshan, Foshan, Guangdong, China; 2 Department of Obstetrics, Southern Medical University Affiliated Maternal and Child Health Hospital of Foshan, Foshan, Guangdong, China; 3 Medical Administration Division, Affiliated Huadu Hospital, Southern Medical University (People’s Hospital of Huadu District), Guangzhou, Guangdong, China

## Abstract

**Importance:**

The existing gestational weight gain (GWG) recommendations for twin pregnancies are lacking for underweight individuals and are not stratified by obesity class.

**Objective:**

To identify optimal GWG ranges associated with reduced adverse perinatal outcomes stratified by prepregnancy body mass index (BMI) categories in twin pregnancies.

**Design, Setting and Participants:**

This population-based cohort study of twin pregnancies using data from the National Center for Health Statistics was conducted between January 1, 2014, and December 31, 2018. Statistical analysis was performed from October 24, 2021, to May 7, 2022. The study population comprised 262 604 individuals between 18 and 45 years of age with live-born twins without congenital malformation between 24 and 42 weeks of gestation. Two approaches were used to determine the optimal GWG ranges: a statistics-based approach calculating IQRs of GWG in a low-risk population, and an outcome-based approach identifying GWG thresholds below or above which an adverse perinatal outcome increased.

**Exposure:**

Gestational weight gain.

**Main Outcomes and Measures:**

Preterm birth less than 36 weeks, gestational hypertensive disorders, small for gestational age status, large for gestational age status, and a composite outcome defined as any occurrence of the individual outcomes.

**Results:**

The main sample comprised 200 810 individuals with twin pregnancies (mean [SD] maternal age, 30.4 [5.5] years; 1624 [0.8%] American Indian or Alaska Native, 13 031 [6.5%] Asian or Pacific Islander, 36 423 [18.1%] Black, and 149 732 [74.6%] White; and 137 409 [68.4%] multiparous). In the low-risk subgroup (n = 61 794), the IQRs of the total GWG after 36 weeks of gestation as assessed using a statistics-based approach and based on BMI group were 15.9 to 22.7 kg for underweight, 15.4 to 22.7 kg for normal weight, 12.7 to 22.2 kg for overweight, 10.0 to 20.0 kg for class 1 obesity, 7.7 to 18.1 kg for class 2 obesity, and 5.9 to 16.3 kg for class 3 obesity. The absolute risk of the composite outcome showed U-shaped associations with GWG across BMI categories. The optimal GWG ranges by 36 weeks identified using an outcome-based approach and BMI group were 17.5 to 24.9 kg for underweight, 15.0 to 24.9 kg for normal weight, 15.0 to 24.9 kg for overweight, 10.0 to 19.9 kg for class 1 obesity, 7.5 to 17.4 kg for class 2 obesity, and 5.0 to 9.9 kg for class 3 obesity. The multivariable logistic models assessed using the validation sample (n = 49 275) showed that GWG defined outside those optimal ranges was associated with preterm birth at less than 36 weeks, gestational hypertensive disorders, and small or large for gestational age.

**Conclusions and Relevance:**

This population-based cohort study found that optimal GWG ranges were similar for individuals with underweight and normal weight but decreased with increasing severity of obesity. The current US Institute of Medicine GWG recommendations may be too high for individuals with moderate or severe obesity.

## Key Points

**Question:**

What is the association of gestational weight gain (GWG) with perinatal outcomes among twin pregnancies across prepregnancy body mass index categories in the US?

**Findings:**

In this cohort study of 200 810 individuals using data from the National Center for Health Statistics, composite adverse perinatal outcome risks showed a U-shaped association with GWG across prepregnancy body mass index categories. The optimal GWG range for individuals with underweight was similar to that for individuals with normal weight but decreased with increasing severity of obesity.

**Meaning:**

These findings may inform prenatal counseling regarding optimal GWG for twin pregnancies and suggest the necessity of updating the current US Institute of Medicine guidelines on GWG.

## Teaser

This cohort study uses natality data from the US National Center for Health Statistics to identify optimal gestational weight gain ranges associated with reduced adverse perinatal outcomes for twin pregnancies stratified by maternal prepregnancy body mass index category.

## Introduction

The rate of twin pregnancies has increased from 1.8% to 3.3% in the last 5 decades.^1^ Compared with singleton pregnancies, twin pregnancies are associated with increased risks of perinatal mortality and morbidity among individuals and their infants, such as preterm birth, gestational hypertensive disorders, gestational diabetes, and fetal growth restriction.^2,3,4^

Maternal prepregnancy body mass index (BMI) and gestational weight gain (GWG) are important factors associated with pregnancy outcomes.^5,6,7^ Abundant research on GWG among both singleton and twin pregnancies has indicated that inadequate GWG is associated with small for gestational age (SGA) status and preterm birth, whereas excessive GWG is associated with large for gestational age (LGA) status, gestational hypertensive disorders, gestational diabetes, and cesarean delivery.^8,9,10,11^ Given the increased nutritional demand and higher risks of adverse outcomes among twin pregnancies, GWG is of great interest in clinical and public health.^12^ The guidelines for GWG among singleton pregnancies are well established, whereas those for twin pregnancies are limited. In 2009, the US Institute of Medicine (IOM) offered only provisional recommendations for GWG in individuals with twin pregnancies,^13^ which reflected the IQR of GWG among individuals with twins who had a mean weight higher than 2500 g at term (GWG, 16.8-24.5 kg for individuals with normal weight, 14.1-22.7 kg for overweight, and 11.3-19.1 kg for obesity of any class) using data obtained from a single study. Recommendations are not available for individuals with underweight or for the various classes of obesity. Therefore, it is necessary to provide optimal GWG ranges for individuals in these BMI groups. Thus, this study explored the GWG ranges of individuals with twin pregnancies stratified by maternal prepregnancy BMI based on natality data obtained from a US national database and examined the feasibility of using these ranges to estimate optimal GWG associated with perinatal outcomes.

## Methods

### Study Participants

This population-based cohort study of individuals with twin pregnancies in the US used natality data from the National Center for Health Statistics of the Centers for Disease Control and Prevention. The natality data represent demographic and health data for live births based on information abstracted from birth certificates. In the present study, the data for twins delivered from January 1, 2014, to December 31, 2018, were used for analysis. Data from January 1, 2014, to December 31, 2017 were included in the main study sample, whereas data from 2018 were included in the validation sample. Statistical analysis was performed from October 24, 2021, to May 7, 2022. This study followed the Strengthening the Reporting of Observational Studies in Epidemiology (STROBE) Statement reporting guideline^14^ and was approved by the institutional review board at Southern Medical University Affiliated Maternal and Child Health Hospital of Foshan. The requirement for informed consent was waived because these public data are deidentified, and the National Center for Health Statistics assumed responsibility for ethical clearance of data collection and publication.

**Figure 1.**
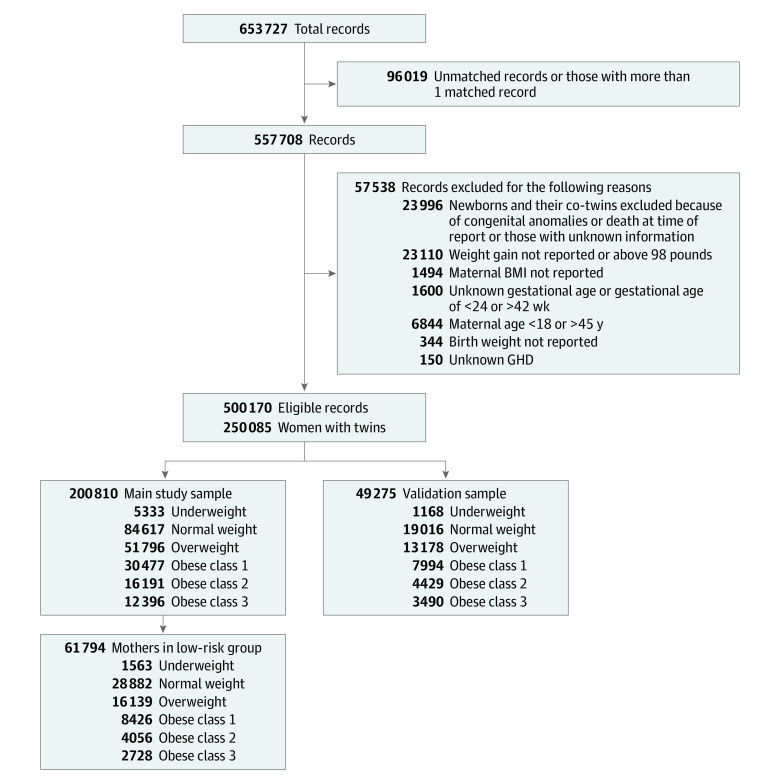
Selection of Eligible Records BMI represents body mass index (calculated as weight in kilograms divided by height in meters squared); and GHD, gestational hypertensive disorder.

Because the data were collected from birth certificates per neonate, we developed an algorithm to match 1 twin with the other, achieving twin pairs based on the variables of parental characteristics and maternal outcomes (eMethods in the Supplement). After excluding records with undetected co-twin status (n = 95 901) or with more than 1 matched record (n = 118), we set the exclusion criteria as follows: (1) maternal age younger than 18 years or older than 45 years; (2) newborns with listed congenital anomalies or deceased at the time of recording; (3) maternal weight gain not reported or higher than 44.5 kg (98 lb); (4) unknown gestational age at birth; (5) gestational age at birth less than 24 weeks or more than 42 weeks; and (6) birth weight not reported. The records of co-twins were also excluded if 1 twin met the exclusion criteria (Figure 1).

### Assessment of BMI and GWG

Maternal prepregnancy BMI was calculated as weight in kilograms divided by height in meters squared based on self-reported prepregnancy height and weight and categorized based on the World Health Organization recommendations as underweight (<18.5), normal weight (18.5-24.9), overweight (25.0-29.9), class 1 obesity (30.0-34.9), class 2 obesity (35.0-39.9) and class 3 obesity (≥40.0).^15^ The GWG rate of individuals was calculated by dividing the GWG by weeks of gestation and is expressed as kilograms per week. The optimal GWG rate ranges were calculated by dividing ranges by 36 weeks for the optimal GWG ranges identified in this study and by 37 weeks for the GWG ranges based on the IOM recommendations (Table). The GWG status was classified as adequate GWG, inadequate GWG, and excessive GWG based on the optimal GWG ranges or on the IOM recommendations. Inadequate GWG was defined as a GWG rate below the optimal range; adequate GWG, as a rate within the range; and excessive GWG, as a rate above the range.

**Table.** Comparison of GWG IQRs Assessed Using the Study Population and IOM Recommendations

BMI category	GWG ranges								IOM recommendations			
	Statistics-based approach				Outcome-based approach							
	Optimal range		GWG rate		Optimal range		GWG rate		IQR		GWG rate	
	kg	lb	kg/wk	lb/wk	kg	lb	kg/wk	lb/wk	kg	lb	kg/wk	lb/wk
Underweight (BMI <18.5)	15.9-22.7	35.1-50.0	0.44-0.63	0.97-1.39	17.5-24.9	38.6-54.9	0.49-0.69	1.07-1.52	ND	ND	ND	ND
Normal weight (BMI 18.5-24.9)	15.4-22.7	34.0-50.0	0.43-0.63	0.94-1.39	15.0-24.9	33.1-54.9	0.42-0.69	0.92-1.52	16.8-24.5	37.0-54.0	0.45-0.66	1.03-1.50
Overweight (BMI 25.0-29.9)	12.7-22.2	28.0-48.9	0.35-0.62	0.78-1.36	15.0-24.9	33.1-54.9	0.42-0.69	0.92-1.52	14.1-22.7	31.1-50.0	0.38-0.61	0.86-1.39
Class 1 obesity (BMI 30.0-34.9)	10.0-20.0	22.0-44.1	0.28-0.56	0.61-1.22	10.0-19.9	22.0-43.9	0.28-0.55	0.61-1.22	11.3-19.1	24.9-42.1	0.31-0.51	0.69-1.17
Class 2 obesity (BMI 35.0-39.9)	7.7-18.1	17.0-39.9	0.21-0.50	0.47-1.11	7.5-17.4	16.5-38.4	0.21-0.48	0.46-1.07	ND	ND	ND	ND
Class 3 obesity (BMI ≥40.0)	5.9-16.3	13.0-35.9	0.16-0.45	0.36-1.00	5.0-9.9	11.0-21.8	0.14-0.28	0.31-0.61	ND	ND	ND	ND

Abbreviations: BMI, body mass index calculated as weight in kilograms divided by height in meters squared; GWG, gestational weight gain; IOM, US Institute of Medicine; ND, not determined.

### Outcomes of Interest

Outcomes of interest included preterm birth less than 36 weeks, gestational hypertensive disorder, SGA status, LGA status, and a composite outcome. Preterm birth less than 36 weeks was determined by the variable “obstetric estimate” of the infant’s gestation, which combined data on the last menstrual period with ultrasonographic confirmation and is considered an accurate estimate.^16^ Gestational hypertensive disorders included pregnancy-induced hypertension and preeclampsia. Because there are no published sex-specific birth weight percentiles for twins in the United States, we developed a sex-specific birth weight reference for twins by replicating the methods of Duryea et al^16^ for their singleton reference (eMethods, eFigure 1, and eTable 1 in the Supplement). Infants with birth weight below the 10th percentile were considered to have SGA status and those above the 90th percentile were considered to have LGA status. The composite outcome was defined as any occurrence of preterm birth less than 36 weeks, gestational hypertensive disorder, SGA status, or LGA status.

### Determination of Optimal GWG

Two approaches were used to determine the optimal GWG ranges for twin pregnancies by BMI category. The first approach (statistics based) was based on a low-risk subgroup that met the following criteria: (1) gestational age at birth between 36 weeks and 42 weeks; (2) both twins at appropriate growth (10th to 90th birth weight percentile); and (3) no prepregnancy diabetes, chronic hypertension, gestational diabetes, gestational hypertensive disorder, or smoking. The optimal GWG ranges for individuals with twin pregnancies by BMI category were calculated as the IQR of total GWG in the low-risk subgroup pregnancies. The second approach (outcome based) was similar to that of Wang et al^17^ and was based on the thresholds of a standardized GWG below or above which the composite adverse outcome increased. Standardized GWG was calculated by multiplying the GWG rate by 36 weeks.

### Statistical Analysis

For the statistics-based approach, the optimal range per BMI category was constructed using the IQR of the total GWG among individuals in the low-risk subgroup. For the outcome-based approach, the adjusted odds ratios (AORs) for the composite outcome were calculated for each standardized GWG group (in 2.5-kg intervals) comparing all other groups using general estimating equation models with binomial distribution and logit link, to address the intertwin correlation. Confounders, including maternal age, race, nulliparity, mode of conception, smoking status, and neonatal sex combination, were taken into account. The optimal GWG range was defined as all standardized GWG groups with a significantly decreased risk of the composite outcome (AOR<1.00 and *P* < .05) and those with nonsignificant ORs (*P* ≥ .05) but between 2 groups with significantly decreased ORs.^17,18^

The external validation of the optimal GWG range was performed using the validation sample of 49 275 individuals. Binary logistic models adjusting for the aforementioned confounders were performed to evaluate the association between GWG status and the individual perinatal outcomes (preterm birth <36 weeks, gestational hypertensive disorders, SGA status, and LGA status), with adequate GWG serving as the reference. The AORs based on the optimal ranges were compared with those based on the IOM guidelines and are shown in forest plots.

All *P* values were 2 tailed, and *P* < .05 was considered statistically significant. All statistical analyses were performed using Stata, version 16.0 (StataCorp).

## Results

### Baseline Characteristics of the Study Population

Among the main sample of 200 810 individuals (mean [SD] maternal age, 30.4 [5.5] years; 1624 [0.8%] American Indian or Alaska Native, 13 031 [6.5%] Asian or Pacific Islander, 36 423 [18.1%] Black, and 149 732 [74.6%] White), 137 409 (68.4%) were multiparous, 181 474 (90.4%) were nonsmokers, and 171 705 (85.5%) had spontaneously conceived; there were 5333 individuals (2.7%) with underweight, 84 617 (42.1%) with normal weight, 51 796 (25.8%) with overweight, 30 477 (15.2%) with class 1 obesity, 16 191 (8.1%) with class 2 obesity, and 12 396 (6.2%) with class 3 obesity. (eTable 2 in the Supplement). In the validation sample of 49 275 individuals (mean [SD] maternal age, 30.5 [5.5] years; 388 [0.8%] American Indian or Alaska Native, 3198 [6.5%] Asian or Pacific Islander, 9653 [19.6%] Black, and 36 036 [73.1%] White), 34 758 (70.5%) were multiparous, 45 129 (91.6%) were nonsmokers, and 43 009 (87.3%) had spontaneously conceived; there were 1168 (2.4%) with underweight, 19 016 (38.6%) with normal weight, 13 178 (26.7%) with overweight, 7994 (16.2%) with class 1 obesity, 4429 (9.0%) with class 2 obesity, and 3490 (7.1%) with class 3 obesity (eTable 3 in the Supplement). The differences between the main sample and the validation sample regarding maternal age, BMI, race and ethnicity, smoking status, marital status, nulliparity, mode of conception (spontaneous conception vs use of fertility-enhancing drugs), gestational diabetes, prepregnancy hypertension, and gestational hypertensive disorder were all statistically significant (eTable 4 in the Supplement).

### Optimal GWG Ranges: Statistics-Based Approach

In the low-risk pregnancy subgroup of 61 794 individuals, there were 1563 individuals with underweight, 28 882 individuals with normal weight, 16 139 individuals with overweight, 8426 individuals with class 1 obesity, 4056 individuals with class 2 obesity, and 2728 individuals with class 3 obesity. Overall, the low-risk subgroup had narrower IQRs for the GWG rate than the high-risk subgroup, and significant differences were found in all BMI groups except the group with overweight (eFigure 2 in the Supplement). The optimal GWG ranges after 36 weeks of gestation obtained by the statistics-based approach were 15.9 to 22.7 kg for underweight, 15.4 to 22.7 kg for normal weight, 12.7 to 22.2 kg for overweight, 10.0 to 20.0 kg for class 1 obesity, 7.7 to 18.1 kg for class 2 obesity, and 5.9 to 16.3 kg for class 3 obesity (Table). Based on the GWG rate, the optimal ranges were similar to the IOM recommendations for the normal weight, overweight, and class 1 obesity groups but were lower than the IOM recommendations for class 2 and 3 obesity groups (Table).

### Optimal GWG Ranges: Outcome-Based Approach

**Figure 2.**
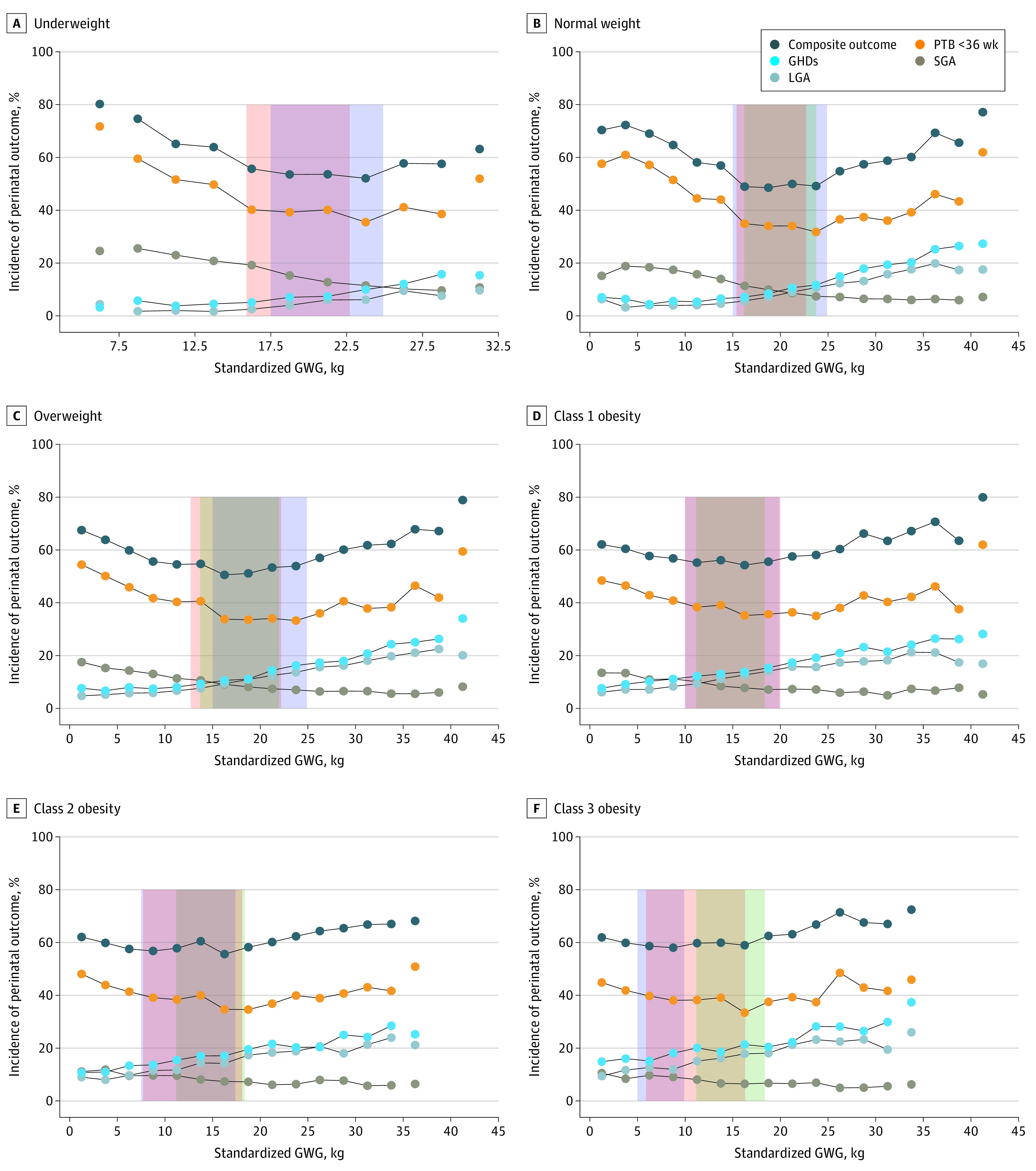
Absolute Risks of Perinatal Outcomes Across Gestational Weight Gain (GWG) Groups by Body Mass Index Category Orange shaded areas represent optimal GWG ranges as assessed by the statistics-based approach; blue shaded areas, optimal GWG ranges as assessed by the outcome-based approach; and green shaded areas, standardized optimal GWG recommended by the US Institute of Medicine (36 weeks’ gestation). Body mass index is calculated as weight in kilograms divided by height in meters squared. GHD represents gestational hypertensive disorder; LGA, large for gestational age status; PTB, preterm birth; and SGA, small for gestational age status.

Figure 2 shows the incidence of the composite outcome and individual morbidities by GWG groups and BMI category, with the numerical results given in eTable 5 in the Supplement. Overall, the absolute risks of preterm birth as well as the composite outcome represented a U-shaped pattern across GWG groups irrespective of BMI category. The incidence of SGA status decreased as GWG increased, whereas the incidence of both gestational hypertensive disorder and LGA status increased as GWG increased.

**Figure 3.**
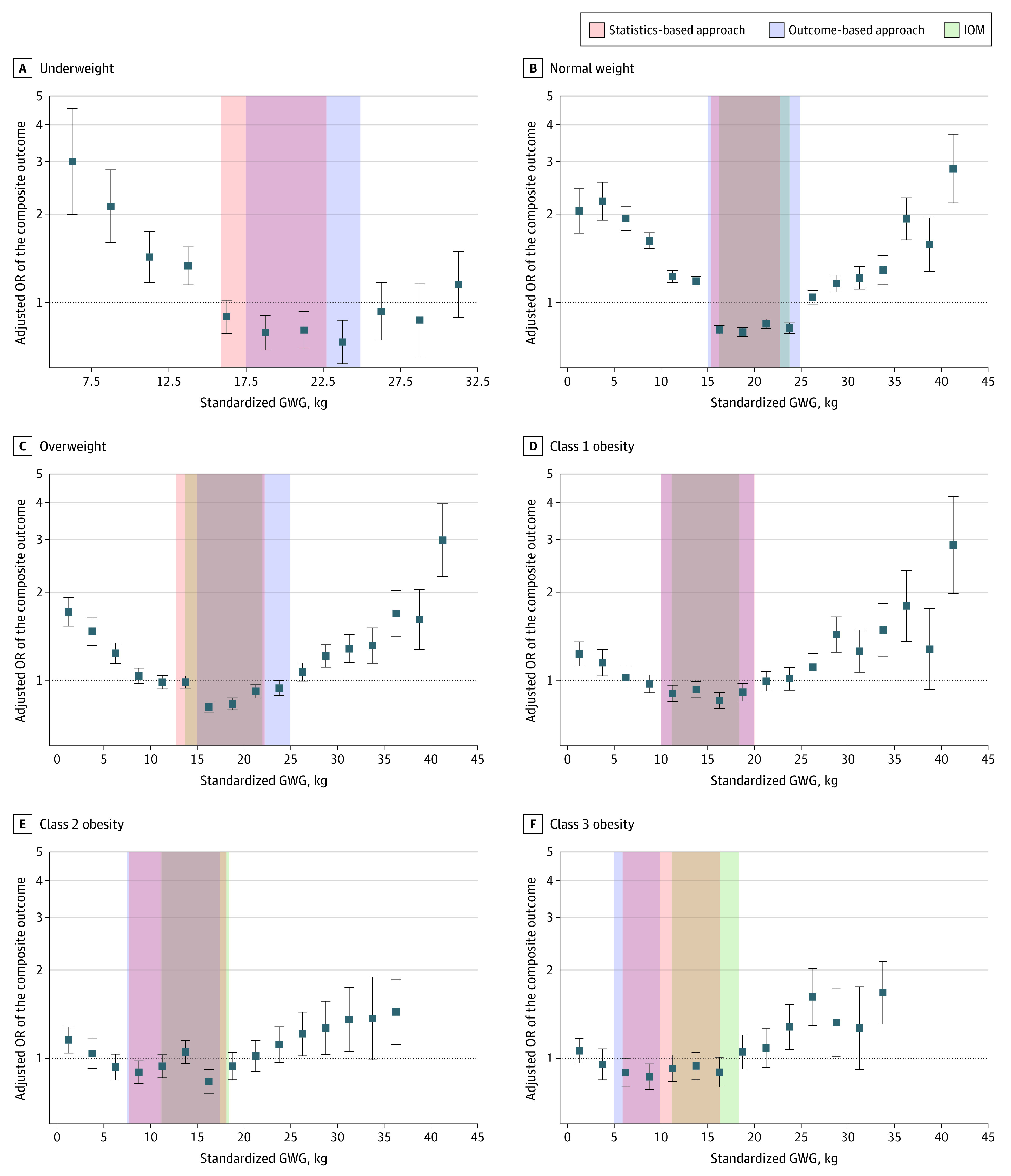
Associations of Gestational Weight Gain (GWG) Groups With the Composite Outcome by Body Mass Index Category Orange shaded areas represent optimal GWG ranges as assessed by the statistics-based approach; blue shaded areas, optimal GWG ranges as assessed by the outcome-based approach; and green shaded areas, standardized optimal GWG recommended by the US Institute of Medicine (IOM; 36 weeks’ gestation). Solid squares represent adjusted odds ratio (OR) for all participants in each GWG group; error bars, 95% CIs.

The association of the GWG group and the composite outcome showed a U-shaped pattern in all BMI categories (Figure 3 and eTable 6 in the Supplement). The optimal GWG ranges after 36 weeks of gestation obtained by the outcome-based approach were 17.5 to 24.9 for the underweight group, 15.0 to 24.9 kg for normal weight, 15.0 to 24.9 kg for overweight, 10.0 to 19.9 kg for class 1 obesity, 7.5 to 17.4 kg for class 2 obesity, and 5.0 to 9.9 kg for class 3 obesity (Table). The optimal ranges were similar to the IOM recommendations for all groups except class 2 and 3 obesity groups (Table).

**Figure 4.**
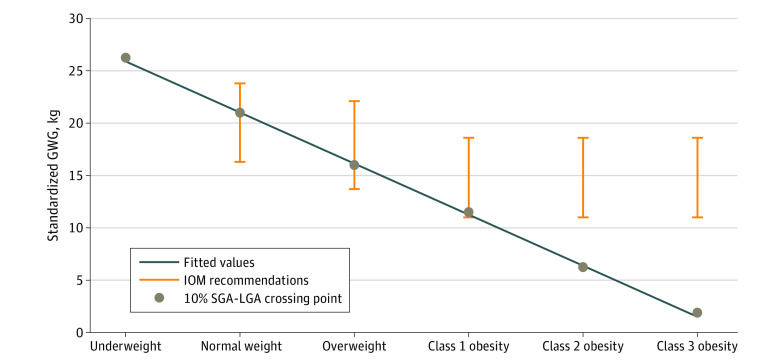
Crossing Points for the Small for Gestational Age (SGA) and Large for Gestational Age (LGA) Curves by Body Mass Index Category Solid circles represent the crossing points; bars indicate optimal gestational weight gain (GWG) ranges recommended by the US Institute of Medicine (IOM). The line indicates the linear fit of the crossing points: optimal GWG (in kg) = (−0.932 × prepregnancy body mass index) + 41.5. Body mass index is calculated as weight in kilograms divided by height in meters squared.

Figure 2 shows that the crossing of the SGA and LGA curves for the incidence of the composite outcome and individual morbidities by GWG groups and BMI categories at 10% shifted by maternal BMI (26.3 kg for underweight, 21.0 kg for normal weight, 16.0 kg for overweight, 11.5 kg for class 1 obesity, 6.3 kg for class 2 obesity, and 1.9 kg for class 3 obesity). These unexpected shifts were absolutely linear using the following calculation: GWG (in kg) = (−0.932 × prepregnancy BMI) + 41.5 (Figure 4). Using that calculator, the GWG for individuals with newborns of appropriate size for their gestational age ranged from 24.7 to 26.6 kg for the underweight group, 19.1 to 23.8 kg for normal weight, 14.5 to 18.2 kg for overweight, 9.8 to 13.5 kg for class 1 obesity, 5.2 to 8.9 kg for class 2 obesity, and −1.4 to 4.2 kg for class 3 obesity (eTable 7 in the Supplement).

### Validation of the Optimal GWG Ranges

The proportions of the GWG status classified by the optimal ranges as assessed by the statistics-based approach, the outcome-based approach, and the IOM recommendations in the validation sample by BMI category are given in eFigure 3 in the Supplement. The AORs of inadequate and excessive GWG for perinatal outcomes, respectively, which were compared between the optimal ranges identified in this study and the IOM recommendations, are given in eFigure 4 and eFigure 5 in the Supplement. Inadequate GWG was associated with increased preterm birth and decreased LGA status in all BMI groups except class 3 obesity using ranges as assessed by the outcome-based approach. Increased AORs for SGA status were found in all BMI groups except class 3 obesity when using the optimal ranges identified in this study. Excessive GWG, whether defined by the optimal ranges identified in this study or the IOM recommendations, was associated with increased gestational hypertensive disorder. Increased LGA status was found in all BMI groups except underweight.

## Discussion

This cohort study of a large population identified optimal GWG ranges associated with reduced adverse perinatal outcomes by BMI category for individuals with twin pregnancies between 36 and 42 weeks of gestation as assessed by a statistics-based approach and an outcome-based approach. For both approaches, the optimal GWG range for individuals with underweight was similar to that for individuals with normal weight. Furthermore, the optimal ranges decreased with increasing severity of obesity. The optimal GWG ranges for the class 2 and 3 obesity groups as assessed by both approaches were lower than those given in the IOM recommendations.

The IOM does not provide optimal GWG recommendations for individuals with underweight who had twin pregnancies because there is a paucity of data available to inform guidelines for this population. Although a previous study by Lal and Kominiarek^19^ evaluated GWG in individuals with underweight using the IOM recommendation for individuals with normal weight and twin pregnancies, the authors did not explain the rationale for this choice. Using both approaches, we found that the optimal GWG ranges for individuals with underweight were similar to the IOM recommendations for individuals with normal weight. This result corroborates a previous finding that individuals with twin gestations and underweight or normal weight had similar GWG had similar perinatal outcomes^20^ and suggests that it is feasible for individuals with underweight and twin pregnancies to adhere to the GWG recommendations for individuals with normal weight prior to the availability of official guidelines for individuals with underweight.

Because both maternal obesity and inappropriate GWG are associated with adverse outcomes in pregnancy and epigenetic consequences across generations,^21,22,23,24,25,26^ recommending a single GWG range for individuals with different classes of obesity is a concern. The results of the present study based on both approaches showed decreasing optimal GWG ranges with increasing severity of obesity, similar to previous studies that calculated optimal GWG for individuals with obesity and singleton pregnancy.^19,21^ In the context of the global obesity epidemic, stratified optimal ranges by obesity class and lower GWG ranges would inform the improved control of GWG and optimize maternal and infant outcomes among individuals with obesity. In addition, both approaches used in the present study indicated that the optimal GWG ranges for class 2 and 3 obesity were lower than the IOM recommendations, especially the lower limits. Lipworth et al^10^ recently reported an optimal GWG range for individuals with obesity and with twin pregnancies lower than the IOM recommendation (9.3-16.3 kg vs 11.3-19.1 kg).

The unexpected linear change in the 10% crossing points for the SGA curves with the LGA curves assessing the incidence of the composite outcome and individual morbidities for each category of rising prepregnancy BMI among twin pregnancies was similar to the finding in singleton pregnancies in the study by Robillard et al.^27^ Those authors suggested that the GWG corresponding to this 10% crossing point of the SGA and LGA curves be called *Maternal Fetal Corpulence Symbiosi*s and that this was the GWG at which individuals could expect to deliver newborns at an appropriate size for their gestational age. Their calculator for optimal GWG in kilograms for individuals with singleton pregnancies was (−1.2 × prepregnancy BMI) + 42. Using that calculator, Robillard et al^27^ concluded that the IOM 2009 recommendations are adequate for individuals with singleton pregnancies and with normal weight or overweight but not with underweight or obesity (eTable 7 in the Supplement). On the basis of the results obtained using our calculator, the IOM GWG recommendations may be too high for achieving twins with appropriate size for gestational age among individuals with class 2 or 3 obesity. Furthermore, our calculator offers optimal GWG for individuals with underweight, which the IOM recommendations did not establish (eTable 7 in the Supplement). Robillard et al^28^ recently emphasized that the optimal GWG calculated from a birth weight perspective may lower the incidence of late onset preeclampsia by 40% among individuals with obesity. However, although the optimal GWG ranges for individuals with extreme obesity may reduce adverse outcomes associated with high GWG, such as gestational hypertensive disorders, they may not reduce adverse outcomes associated with low or U-shaped GWG, such as preterm birth. A balance in the risks and benefits among individuals and offspring is needed not only between GWG and maternal BMI but also in the GWG itself.

Although the optimal GWG ranges in the present study were calculated based on a composite outcome, the validation analyses were performed based on individual morbidities, which inform prenatal counseling. Our results suggest that inadequate GWG may be associated with increased preterm birth and SGA status, whereas excessive GWG may be associated with increased gestational hypertensive disorders and LGA status, findings consistent with most relevant reports,^19,29,30,31^ suggesting that our optimal ranges are feasible.

### Limitations

This study had some limitations. First, some bias regarding the retrospective nature of the present study, such as the self-reported prepregnancy weight of individuals, cannot be avoided, despite the acceptable reporting error, as described previously.^32^ Moreover, the database did not include information on chorionicity. We used the neonatal sex combination as a proxy, which is considered suboptimal.^33^ Second, we used an algorithm that was relatively stricter than that used in a previous study^34^ to achieve twin pairs to avoid mismatch. As a result, there was a lower twinning detection rate than in that previous study (85.3% vs 90.0%). Third, the use of a standardized GWG and the assessment of the GWG status based on the assumption of a linear GWG may introduce bias because GWG is lower in the first and late third trimesters of pregnancy. Fourth, gestational diabetes was not included in outcomes of interest in the outcome-based approach because weight control is likely to be advised to individuals with gestational diabetes and consequently could have caused bias in the results.

## Conclusions

This population-based cohort study found that for twin pregnancies, the optimal GWG range associated with reduced adverse perinatal outcomes for individuals with underweight was similar to that for individuals with normal weight but decreased with increasing severity of obesity. The current IOM GWG recommendations may be too high for individuals with moderate or severe obesity.

## Notes

Supplement.
eMethods.
**eTable 1.** Sex-Specific Birthweight Percentiles in Twins **eTable 2.** Characteristics and Outcomes of Main Study Sample **eTable 3.** Characteristics and Outcomes of Validation Sample **eTable 4.** Comparison Between the Main Sample and Validation Sample **eTable 5.** Incidence of Outcomes by Standardized GWG Category and BMI Category (Used as Main Study Sample) **eTable 6.** Association of GWG Category and Composite Outcome by Maternal BMI Category (Used as Main Study Sample) **eTable 7.** Comparison Between IOM 2009 and Current Optimal GWG Ranges and GWG Corresponding to Maternal Fetal Corpulence Symbiosis (MFCS) **eFigure 1.** Sex-Specific Birthweight Percentile Curves in Twins (Used as Both Main Study Sample and Validation Sample) **eFigure 2.** The Median and Interquartile Range (IQR) of GWG Rate in Low-risk and High-risk Subgroups Stratified by BMI Category (Used as Main Study Sample) **eFigure 3.** Proportions of Inadequate, Adequate and Excessive GWG Defined Based on the Optimal Ranges (Statistical-Based and Outcome-Based Approach) and the IOM Recommendations by Prepregnancy BMI Category (Used as Validation Sample) **eFigure 4.** Adjusted Odd Ratios (aORs) for Perinatal Outcomes of Inadequate GWG (Used as Validation Sample) **eFigure 5.** Adjusted Odd Ratios (aORs) for Perinatal Outcomes of Excessive GWG (Used as Validation Sample)
