# Supplementary material for: Association of Optimal Gestational Weight Gain Ranges With Perinatal Outcomes Across Body Mass Index Categories in Twin Pregnancies
Source: JAMA Netw Open. 2022 Jul 19;5(7):e2222537. doi: 10.1001/jamanetworkopen.2022.22537 (PMC9297120; doi:10.1001/jamanetworkopen.2022.22537)
Supplement: Supplement. — eMethods. eTable 1. Sex-Specific Birthweight Percentiles in Twins eTable 2. Characteristics and Outcomes of Main Study Sample eTable 3. Characteristics and Outcomes of Validation Sample eTable 4. Comparison Between the Main Sample and Validation Sample eTable 5. Incidence of Outcomes by Standardized GWG Category and BMI Category (Used as Main Study Sample) eTable 6. Association of GWG Category and Composite Outcome by Maternal BMI Category (Used as Main Study Sample) eTable 7. Comparison Between IOM 2009 and Current Optimal GWG Ranges and GWG Corresponding to Maternal Fetal Corpulence Symbiosis (MFCS) eFigure 1. Sex-Specific Birthweight Percentile Curves in Twins (Used as Both Main Study Sample and Validation Sample) eFigure 2. The Median and Interquartile Range (IQR) of GWG Rate in Low-risk and High-risk Subgroups Stratified by BMI Category (Used as Main Study Sample) eFigure 3. Proportions of Inadequate, Adequate and Excessive GWG Defined Based on the Optimal Ranges (Statistical-Based and Outcome-Based Approach) and the IOM Recommendations by Prepregnancy BMI Category (Used as Validation Sample) eFigure 4. Adjusted Odd Ratios (aORs) for Perinatal Outcomes of Inadequate GWG (Used as Validation Sample) eFigure 5. Adjusted Odd Ratios (aORs) for Perinatal Outcomes of Excessive GWG (Used as Validation Sample) [file jamanetwopen-e2222537-s001.pdf]

Supplementary Online Content

Lin D, Huang X, Fan D, et al. Association of optimal gestational weight gain ranges with perinatal outcomes across body mass index categories in twin pregnancies. *JAMA Netw Open*. 2022;5(7):e2222537. doi:10.1001/jamanetworkopen.2022.22537

eMethods.
eTable 1. Sex-Specific Birthweight Percentiles in Twins
eTable 2. Characteristics and Outcomes of Main Study Sample
eTable 3. Characteristics and Outcomes of Validation Sample
eTable 4. Comparison Between the Main Sample and Validation Sample
eTable 5. Incidence of Outcomes by Standardized GWG Category and BMI Category (Used as Main Study Sample)
eTable 6. Association of GWG Category and Composite Outcome by Maternal BMI Category (Used as Main Study Sample)
eTable 7. Comparison Between IOM 2009 and Current Optimal GWG Ranges and GWG Corresponding to Maternal Fetal Corpulence Symbiosis (MFCS)
eFigure 1. Sex-Specific Birthweight Percentile Curves in Twins (Used as Both Main Study Sample and Validation Sample)
eFigure 2. The Median and Interquartile Range (IQR) of GWG Rate in Low-Risk and High-Risk Subgroups Stratified by BMI Category (Used as Main Study Sample)
eFigure 3. Proportions of Inadequate, Adequate and Excessive GWG Defined Based on the Optimal Ranges (Statistical-Based and Outcome-Based Approach) and the IOM Recommendations by Prepregnancy BMI Category (Used as Validation Sample)
eFigure 4. Adjusted Odd Ratios (aORs) for Perinatal Outcomes of Inadequate GWG (Used as Validation Sample)
eFigure 5. Adjusted Odd Ratios (aORs) for Perinatal Outcomes of Excessive GWG (Used as Validation Sample)

This supplementary material has been provided by the authors to give readers additional information about their work.

© 2022 Lin D et al. *JAMA Network Open.*

eMethods.

The variables for developing the algorithm to achieve twin pairs included dob_yy, mager, mbstate_rec, restatus, mrace31, mrace6, mrace15, mbrace, mhisp_r, mracehisp, dmar, meduc, fagecomb, frace31, frace6, frace15, fbrace, fhisp_r, fracehisp, feduc, precare, previs, cig_0, cig_1, cig_2, cig_3, m_ht_in, bmi, pwgt_r, dwgt_r, wtgain, rf_pdiab, rf_gdiab, rf_phype, rf_ghype, rf_ehype, rf_inftr, rf_fedrg, rf_artec, ip_gon, ip_syph, ip_chlam, ip_hepatb, ip_hepatc, mm_mtr, mm_plac, mm_rupt, mm_uhyst, mm_aicu, pay, dlmp_mm, dlmp_yy and oegest_comb.

Maternal smoking status was classified as "never smoke", "smoke only before pregnancy" and "smoke during pregnancy with/without smoking before pregnancy". The method of conception was classified as spontaneous, fertility-enhancing drugs for ovulation induction (OI) and/or intrauterine insemination (IUI) and assisted reproductive technology (e.g., in vitro fertilization (IVF), gamete intrafallopian transfer (GIFT), and zygote intrafallopian transfer (ZIFT)). Neonatal sex was classified as male-male, male-female and female-female.

The birthweight percentiles were calculated based on a subgroup population that met the following criteria: (1) no known congenital anomalies; (2) gestational age at birth between 24 weeks and 42 weeks; and (3) birthweight between 500 g and 6,000 g. Quantile regression models using cubic splines with 5 knots were used to calculate the 5th, 10th, 50th, 90th and 95th percentile cut points by gestational week and draw growth curves for the male and female twins (eTable 1 and eFigure 1 in the Supplement).

To ensure exact comparison between the IOM recommendations versus the optimal GWG ranges. The IOM recommendations were standardized for 36 weeks of gestation by dividing the ranges by 37 weeks and multiplying 36 weeks (16.3-23.8 kg for normal weight, 13,7-22.1kg for overweight and 11.0-18.6 for obesity).

© 2022 Lin D et al. *JAMA Network Open.*

eTable 1. Sex-Specific Birthweight Percentiles in Twins

		Female							Male				
Weeks of gestation	n	5th	10th	50th	90th	95th		n	5th	10th	50th	90th	95th
24	820	567	583	680	810	873		857	595	624	735	873	910
25	1,127	551	600	750	897	960		1,109	571	638	804	960	1007
26	1,375	573	648	843	1010	1076		1,543	592	686	899	1077	1132
27	1,831	630	724	956	1145	1215		1,895	652	765	1017	1219	1283
28	2,411	716	823	1087	1301	1377		2,434	743	868	1153	1382	1455
29	2,844	822	939	1232	1476	1559		3,016	854	988	1304	1563	1646
30	3,911	952	1073	1389	1663	1753		4,210	992	1129	1469	1758	1850
31	5,665	1094	1218	1556	1861	1960		5,855	1142	1280	1642	1963	2065
32	9,592	1248	1372	1729	2066	2173		9,564	1304	1440	1822	2174	2285
33	13,150	1422	1543	1908	2268	2383		13,170	1490	1619	2010	2388	2504
34	23,365	1589	1708	2087	2475	2598		23,536	1663	1789	2195	2600	2724
35	30,149	1754	1871	2265	2679	2809		30,644	1831	1956	2377	2807	2939
36	48,142	1927	2041	2441	2869	3005		47,751	2011	2131	2558	3005	3140
37	59,115	2070	2187	2608	3060	3200		58,899	2145	2275	2722	3190	3333
38	40,711	2230	2347	2769	3220	3363		41,408	2309	2438	2886	3358	3500
39	3,885	2340	2466	2915	3381	3524		4,030	2400	2549	3023	3506	3654
40	834	2450	2586	3049	3509	3651		820	2496	2663	3150	3629	3777
41	117	2537	2687	3166	3611	3749		119	2565	2756	3258	3724	3869
42	35	2574	2748	3262	3697	3827		35	2565	2799	3333	3787	3934

© 2022 Lin D et al. *JAMA Network Open.*

eTable 2. Characteristics and Outcomes of Main Study Sample (n=200,810)

Characteristics or outcomes	Total (200,810)	Underweight (n=5,333)	Normal weight (n=84,617)	Overweight (n=51,796)	Obesity I (n=30,477)	Obesity II (n=16,191)	Obesity III (n=12,396)
Maternal age	30.4±5.5	29.0±5.8	30.7±5.5	30.5±5.6	30.1±5.6	29.8±5.5	29.4±5.3
Race and ethnicity							
White	149,732 (74.6)	3,803 (71.3)	66,194 (78.2)	38,678 (74.7)	21,811 (71.6)	11,297 (69.8)	7,949 (64.1)
Black	36,423 (18.1)	668 (12.5)	10,263 (12.1)	9,750 (18.8)	7,242 (23.8)	4,358 (26.9)	4,142 (33.4)
American Indian or Alaskan Native	1,624 (0.8)	25 (0.5)	470 (0.6)	467 (0.9)	327 (1.1)	182 (1.1)	153 (1.2)
Asian or Pacific Islander	13,031 (6.5)	837 (15.7)	7,690 (9.1)	2,901 (5.6)	1,097 (3.6)	354 (2.2)	152 (1.2)
Smoking status							
Never smoke	181,474 (90.4)	4,552 (85.4)	77,586 (91.7)	47,009 (90.8)	27,181 (89.2)	14,305 (88.4)	10,841 (87.5)
Only smoke before pregnancy	4,768 (2.4)	119 (2.2)	1,656 (2.0)	1,197 (2.3)	890 (2.9)	491 (3.0)	415 (3.4)
Only smoke during pregnancy	198 (0.1)	10 (0.2)	65 (0.1)	52 (0.1)	32 (0.1)	17 (0.1)	22 (0.2)
Smoke before and during pregnancy	12,944 (6.5)	600 (11.3)	4,711 (5.6)	3,178 (6.1)	2,159 (7.1)	1,261 (7.8)	1,035 (8.4)

© 2022 Lin D et al. *JAMA Network Open.*

Unknown	1,426 (0.7)	52 (1.0)	599 (0.7)	360 (0.7)	215 (0.7)	117 (0.7)	83 (0.7)
Marital status							
Married	132,430 (66.0)	3,362 (63)	60,819 (71.9)	33,995 (65.6)	18,509 (60.7)	9,302 (57.5)	6,443 (52.0)
Unmarried	62,631 (31.2)	1,821 (34.2)	21,280 (25.2)	16,203 (31.3)	11,109 (36.5)	6,526 (40.3)	5,692 (45.9)
Unknown	5,749 (2.9)	150 (2.8)	2,518 (3.0)	1,598 (3.1)	859 (2.8)	363 (2.2)	261 (2.1)
Nulliparity							
No	137,409 (68.4)	3,225 (60.5)	53,827 (63.6)	36,423 (70.3)	22,602 (74.2)	11,984 (74)	9,348 (75.4)
Yes	61,748 (30.8)	2,051 (38.5)	30,122 (35.6)	14,946 (28.9)	7,634 (25.1)	4,055 (25)	2,940 (23.7)
Unknown	1,653 (0.8)	57 (1.1)	668 (0.8)	427 (0.8)	241 (0.8)	152 (0.9)	108 (0.9)
Method of conception							
Spontaneous	171,705 (85.5)	4,653 (87.3)	69,956 (82.7)	44,432 (85.8)	26,782 (87.9)	14,469 (89.4)	11,413 (92.1)
Fertility-enhancing drugs for OI and/or IUI	8,431 (4.2)	173 (3.2)	4,049 (4.8)	2,065 (4.0)	1,092 (3.6)	615 (3.8)	437 (3.5)
Assisted reproductive technology	18,737 (9.3)	476 (8.9)	9,620 (11.4)	4,822 (9.3)	2,345 (7.7)	992 (6.1)	482 (3.9)
Unknown	1,937 (1.0)	31 (0.6)	992 (1.2)	477 (0.9)	258 (0.9)	115 (0.7)	64 (0.5)
Neonatal sex combination							
Male-Male	64,537 (32.1)	1,807 (33.9)	27,852 (32.9)	16,797 (32.4)	9,559 (31.4)	4,861 (30.0)	3,661 (29.5)

© 2022 Lin D et al. *JAMA Network Open.*

Female-female	63,770 (31.8)	1,819 (34.1)	27,280 (32.2)	16,378 (31.6)	9,633 (31.6)	4,935 (30.5)	3,725 (30.1)
Male-female	72,503 (36.1)	1,707 (32)	29,485 (34.9)	18,621 (36.0)	11,285 (37.0)	6,395 (39.5)	5,010 (40.4)
Pre-pregnancy diabetes							
No	199,006 (99.1)	5,315 (99.7)	84,271 (99.6)	51,424 (99.3)	30,084 (98.7)	15,851 (97.9)	12,061 (97.3)
Yes	1,804 (0.9)	18 (0.3)	346 (0.4)	372 (0.7)	393 (1.3)	340 (2.1)	335 (2.7)
GDM							
No	184,783 (92.0)	5,102 (95.7)	79,911 (94.4)	47,669 (92.0)	27,287 (89.5)	14,173 (87.5)	10,641 (85.8)
Yes	16,027 (8.0)	231 (4.3)	4,706 (5.6)	4,127 (8.0)	3,190 (10.5)	2,018 (12.5)	1,755 (14.2)
Pre-pregnancy hypertension							
No	195,821 (97.5)	5,304 (99.5)	83,772 (99.0)	50,836 (98.2)	29,382 (96.4)	15,295 (94.5)	11,232 (90.6)
Yes	4,989 (2.5)	29 (0.5)	845 (1.0)	960 (1.9)	1,095 (3.6)	896 (5.5)	1,164 (9.4)
GHDs							
No	176,824 (88.1)	4,949 (92.8)	76,602 (90.5)	45,607 (88.0)	26,148 (85.8)	13,540 (83.6)	9,978 (80.5)
Yes	23,986 (11.9)	384 (7.2)	8,015 (9.5)	6,189 (12.0)	4,329 (14.2)	2,651 (16.4)	2,418 (19.5)
PTB <36 weeks							
No	122,982 (61.2)	2,994 (56.1)	52,197 (61.7)	32,063 (61.9)	18,517 (60.8)	9,749 (60.2)	7,462 (60.2)
Yes	77,828 (38.8)	2,339 (43.9)	32,420 (38.3)	19,733 (38.1)	11,960 (39.2)	6,442 (39.8)	4,934 (39.8)

© 2022 Lin D et al. *JAMA Network Open.*

SGA (calculated as per newborn)							
No	361,386 (90.0)	8,885 (83.3)	150,876 (89.2)	93,723 (90.5)	55,512 (91.1)	29,581 (91.4)	22,809 (92.0)
Yes	40,234 (10.0)	1,781 (16.7)	18,358 (10.9)	9,869 (9.5)	5,442 (8.9)	2,801 (8.7)	1,983 (8.0)
LGA (calculated as per newborn)							
No	362,171 (90.2)	10,205 (95.7)	156,241 (92.3)	92,975 (89.8)	53,757 (88.2)	27,974 (86.4)	21,019 (84.8)
Yes	39,449 (9.8)	461 (4.3)	12,993 (7.7)	10,617 (10.3)	7,197 (11.8)	4,408 (13.6)	3,773 (15.2)
OI, ovulation induction; IUI, intrauterine insemination; GDM, gestational diabetes mellitus; GHDs, gestational hypertensive disorders; PTB, preterm birth; SGA, small for gestational age; LGA, large for gestational age.							

© 2022 Lin D et al. *JAMA Network Open.*

eTable 3. Characteristics and Outcomes of Validation Sample (n=49,275)

Characteristics or outcomes	Total (49,275)	Underweight (n=1,168)	Normal weight (n=19,016)	Overweight (n=13,178)	Obesity I (n=7,994)	Obesity II (n=4,429)	Obesity III (n=3,490)
Maternal age	30.5±5.5	29.1±5.8	30.8±5.5	30.6±5.4	30.3±5.5	29.9±5.4	29.7±5.1
Race and ethnicity							
White	36,036 (73.1)	801 (68.6)	14,609 (76.8)	9,662 (73.3)	5,670 (70.9)	3,015 (68.1)	2,279 (65.3)
Black	9,653 (19.6)	189 (16.2)	2,526 (13.3)	2,596 (19.7)	1,944 (24.3)	1,264 (28.5)	1,134 (32.5)
American Indian or Alaskan Native	388 (0.8)	6 (0.5)	105 (0.6)	109 (0.8)	76 (1)	51 (1.2)	41 (1.2)
Asian or Pacific Islander	3,198 (6.5)	172 (14.7)	1,776 (9.3)	811 (6.2)	304 (3.8)	99 (2.2)	36 (1)
Smoking status							
Never smoke	45,129 (91.6)	1,027 (87.9)	17,640 (92.8)	12,110 (91.9)	7,245 (90.6)	3,979 (89.8)	3,128 (89.6)
Only smoke before pregnancy	1,009 (2.1)	22 (1.9)	296 (1.6)	262 (2)	199 (2.5)	133 (3.0)	97 (2.8)
Only smoke during pregnancy	47 (0.1)	1 (0.1)	15 (0.1)	13 (0.1)	11 (0.1)	3 (0.1)	4 (0.1)
Smoke before and during pregnancy	2,914 (5.9)	111 (9.5)	1004 (5.3)	747 (5.7)	508 (6.4)	297 (6.7)	247 (7.1)
Unknown	176 (0.4)	7 (0.6)	61 (0.3)	46 (0.4)	31 (0.4)	17 (0.4)	14 (0.4)
Marital status							
Married	28,683 (58.2)	592 (50.7)	12,027 (63.3)	7,738 (58.7)	4,392 (54.9)	2,244 (50.7)	1,690 (48.4)
Unmarried	1,5065 (30.6)	413 (35.4)	4,710 (24.8)	3,906 (29.6)	2,769 (34.6)	1,745 (39.4)	1,522 (43.6)
Unknown	5,527 (11.2)	163 (14.0)	2,279 (12.0)	1,534 (11.6)	833 (10.4)	440 (9.9)	278 (8.0)
Nulliparity							
No	34,758 (70.5)	731 (62.6)	12,593 (66.2)	9,393 (71.3)	5,976 (74.8)	3,371 (76.1)	2,694 (77.2)
Yes	14,298 (29)	429 (36.7)	6,333 (33.3)	3,735 (28.3)	1,976 (24.7)	1,038 (23.4)	787 (22.6)
Unknown	219 (0.4)	8 (0.7)	90 (0.5)	50 (0.4)	42 (0.5)	20 (0.5)	9 (0.3)
Method of conception							
Spontaneous	43,009 (87.3)	1,038 (88.9)	16,154 (85.0)	11,487 (87.2)	7,095 (88.8)	4,009 (90.5)	3,226 (92.4)
Fertility-enhancing drugs for OI and/or IUI	1,877 (3.8)	36 (3.1)	799 (4.2)	509 (3.9)	284 (3.6)	142 (3.2)	107 (3.1)
Assisted reproductive technology	4,119 (8.4)	90 (7.7)	1,959 (10.3)	1,096 (8.3)	579 (7.2)	257 (5.8)	138 (4.0)
Unknown	270 (0.6)	4 (0.3)	104 (0.6)	86 (0.7)	36 (0.5)	21 (0.5)	19 (0.5)
Neonatal sex combination							
Male-Male	15,928 (32.3)	416 (35.6)	6,307 (33.2)	4,233 (32.1)	2,574 (32.2)	1,361 (30.7)	1,037 (29.7)
Female-female	15,828 (32.1)	425 (36.4)	6,261 (32.9)	4,284 (32.5)	2,487 (31.1)	1,337 (30.2)	1,034 (29.6)

© 2022 Lin D et al. *JAMA Network Open.*

Male-female	17,519 (35.6)	327 (28)	6,448 (33.9)	4,661 (35.4)	2,933 (36.7)	1,731 (39.1)	1,419 (40.7)
Pre-pregnancy diabetes							
No	48,826 (99.1)	1,166 (99.8)	18,940 (99.6)	13,075 (99.2)	7,899 (98.8)	4,351 (98.2)	3,395 (97.3)
Yes	449 (0.9)	2 (0.2)	76 (0.4)	103 (0.8)	95 (1.2)	78 (1.8)	95 (2.7)
GDM							
No	44,727 (90.8)	1,111 (95.1)	17,740 (93.3)	12,025 (91.3)	7,081 (88.6)	3,824 (86.3)	2,946 (84.4)
Yes	4,548 (9.2)	57 (4.9)	1276 (6.7)	1153 (8.8)	913 (11.4)	605 (13.7)	544 (15.6)
Pre-pregnancy hypertension							
No	47,807 (97.0)	1,156 (99.0)	18,785 (98.8)	12,913 (98.0)	7,688 (96.2)	4,156 (93.8)	3,109 (89.1)
Yes	1,468 (3.0)	12 (1.0)	231 (1.2)	265 (2.0)	306 (3.8)	273 (6.2)	381 (10.9)
GHDs							
No	42,542 (86.3)	1,064 (91.1)	16,960 (89.2)	11,466 (87)	6,688 (83.7)	3,602 (81.3)	2,762 (79.1)
Yes	6,733 (13.7)	104 (8.9)	2,056 (10.8)	1,712 (13)	1,306 (16.3)	827 (18.7)	728 (20.9)
PTB <36 weeks							
No	29,943 (60.8)	611 (52.3)	11,706 (61.6)	8,110 (61.5)	4,776 (59.7)	2,646 (59.7)	2,094 (60.0)
Yes	19,332 (39.2)	557 (47.7)	7,310 (38.4)	5,068 (38.5)	3,218 (40.3)	1,783 (40.3)	1,396 (40.0)
SGA (calculated as per newborn)							
No	88,881 (90.2)	1,990 (85.2)	33,831 (89)	23,910 (90.7)	14,555 (91.0)	8,122 (91.7)	6,473 (92.7)
Yes	9,669 (9.8)	346 (14.8)	4201 (11.1)	2,446 (9.3)	1,433 (9.0)	736 (8.3)	507 (7.3)
LGA (calculated as per newborn)							
No	88,682 (90.0)	2,246 (96.2)	35,048 (92.2)	23,671 (89.8)	14,130 (88.4)	7,668 (86.6)	5,919 (84.8)
Yes	9,868 (10.0)	90 (3.9)	2,984 (7.9)	2,685 (10.2)	1,858 (11.6)	1,190 (13.4)	1,061 (15.2)

OI, ovulation induction; IUI, intrauterine insemination; GDM, gestational diabetes mellitus; GHDs, gestational hypertensive disorders; PTB, preterm birth; SGA, small for gestational age; LGA, large for gestational age.

© 2022 Lin D et al. *JAMA Network Open.*

eTable 4. Comparison Between the Main Sample and Validation Sample

	Main Sample (n= 200,810)	Validation Sample (n= 49,275)	*P*-value
Characteristics or outcomes			
Maternal age	30.4±5.5	30.5±5.5	<0.001
BMI category			
Underweight	5,333 (2.7)	1,168 (2.4)	<0.001
Normal-weight	84,617 (42.1)	19,016 (38.6)	
Overweight	51,796 (25.8)	13,178 (26.7)	
Obese Class I	30,477 (15.2)	7,994 (16.2)	
Obese Class II	16,191 (8.1)	4,429 (9.0)	
Obese Class III	12,396 (6.2)	3,490 (7.1)	
Race and ethnicity			
White	149,732 (74.6)	36,036 (73.1)	<0.001
Black	36,423 (18.1)	9,653 (19.6)	
American Indian or Alaskan Native	1,624 (0.8)	388 (0.8)	
Asian or Pacific Islander	13,031 (6.5)	3,198 (6.5)	
Smoking status			
Never smoke	181,474 (90.4)	45,129 (91.6)	<0.001
Only smoke before pregnancy	4,768 (2.4)	1,009 (2.1)	
Only smoke during pregnancy	198 (0.1)	47 (0.1)	
Smoke before and during pregnancy	12,944 (6.5)	2,914 (5.9)	
Unknown			
Marital status			
Married	132,430 (66.0)	28,683 (58.2)	<0.001
Unmarried	62,631 (31.2)	15,065 (30.6)	
Unknown	5,749 (2.9)	5,527 (11.2)	
Nulliparity			
No	137,409 (68.4)	34,758 (70.5)	<0.001
Yes	61,748 (30.8)	14,298 (29.0)	
Unknown	1,653 (0.8)	219 (0.5)	
Method of conception			
Spontaneous	171,705 (85.5)	43,009 (87.3)	<0.001
Fertility-enhancing drugs for OI and/or IUI	8,431 (4.2)	1,877 (3.8)	
Assisted reproductive technology	18,737 (9.3)	4,119 (8.4)	
Unknown	1,937 (1.0)	270 (0.6)	
Neonatal sex combination			
Male-Male	64,537 (32.1)	15,928 (32.3)	0.066
Female-female	63,770 (31.8)	15,828 (32.1)	
Male-female	72,503 (36.1)	17,519 (35.6)	
Pre-pregnancy diabetes	1,804 (0.9)	449 (0.9)	0.787
GDM	16,027 (8.0)	4,548 (9.2)	<0.001
Pre-pregnancy hypertension	4,989 (2.5)	1,468 (3.0)	<0.001
GHDs	23,986 (11.9)	6,733 (13.7)	<0.001
PTB <36 weeks	77,828 (38.8)	19,332 (39.2)	0.052
PTB <34 weeks	34,859 (17.4)	8,464 (17.2)	0.338
PTB <32 weeks	16,637 (8.3)	3,954 (8.0)	0.059

© 2022 Lin D et al. *JAMA Network Open.*

SGA (calculated as per newborn)	40,234 (10.0)	9,669 (9.8)	0.052
LGA (calculated as per newborn)	39,449 (9.8)	9,868 (10.0)	0.072

© 2022 Lin D et al. *JAMA Network Open.*

eTable 5. Incidence of Outcomes by Standardized GWG Category and BMI Category (Used as Main Study Sample)

	Composite Outcome	PTB<36 weeks	GHDs	SGA	LGA
Standardized GWG (kg)	Cases [n (%)]*	Cases [n (%)]	Cases [n (%)]	Cases [n (%)]*	Cases [n (%)]*
Underweight (n=5,333)					
< 7.5	199 (80.2)	89 (71.8)	4 (3.2)	61 (24.6)	11 (4.4)
7.5-9.9	336 (74.7)	134 (59.6)	13 (5.8)	115 (25.6)	8 (1.8)
10.0-12.4	512 (65.1)	203 (51.7)	15 (3.8)	181 (23.0)	16 (2.0)
12.5-14.9	982 (63.9)	382 (49.7)	35 (4.6)	320 (20.8)	26 (1.7)
15.0-17.4	1109 (55.7)	400 (40.2)	51 (5.1)	383 (19.3)	51 (2.6)
17.5-19.9	957 (53.6)	351 (39.3)	63 (7.1)	274 (15.3)	72 (4.0)
20.0-22.4	780 (53.7)	292 (40.2)	54 (7.4)	186 (12.8)	88 (6.1)
22.5-24.9	543 (52.1)	185 (35.5)	52 (10.0)	120 (11.5)	64 (6.1)
25.0-27.4	334 (57.8)	119 (41.2)	35 (12.1)	59 (10.2)	55 (9.5)
27.5-29.9	197 (57.6)	66 (38.6)	27 (15.8)	33 (9.7)	26 (7.6)
≥30.0	287 (63.2)	118 (52.0)	35 (15.4)	49 (10.8)	44 (9.7)
Normal-weight (n=84,617)					
0-2.4	743 (70.4)	304 (57.6)	37 (7.0)	160 (15.2)	67 (6.3)
2.5-4.9	1091 (72.3)	460 (60.9)	48 (6.4)	284 (18.8)	48 (3.2)
5.0-7.4	2393 (69.0)	991 (57.2)	77 (4.4)	637 (18.4)	138 (4.0)
7.5-9.9	4987 (64.7)	1983 (51.5)	215 (5.6)	1342 (17.4)	304 (3.9)
10.0-12.4	7945 (58.1)	3042 (44.5)	363 (5.3)	2149 (15.7)	548 (4.0)
12.5-14.9	12387 (56.9)	4788 (44.0)	707 (6.5)	3029 (13.9)	1001 (4.6)
15.0-17.4	13716 (48.9)	4890 (34.9)	995 (7.1)	3182 (11.4)	1583 (5.7)
17.5-19.9	13323 (48.5)	4667 (34.0)	1156 (8.4)	2725 (9.9)	1938 (7.1)
20.0-22.4	11299 (49.9)	3852 (34.1)	1201 (10.6)	1935 (8.6)	2036 (9.0)
22.5-24.9	8346 (49.2)	2695 (31.7)	991 (11.7)	1254 (7.4)	1815 (10.7)
25.0-27.4	5649 (54.8)	1883 (36.5)	769 (14.9)	732 (7.1)	1271 (12.3)
27.5-29.9	3718 (57.4)	1211 (37.4)	579 (17.9)	416 (6.4)	852 (13.2)
30.0-32.4	2144 (58.7)	658 (36.1)	353 (19.3)	233 (6.4)	575 (15.8)

© 2022 Lin D et al. *JAMA Network Open.*

32.5-34.9	1300 (60.1)		424 (39.2)		219 (20.3)		130 (6.0)		381 (17.6)
35.0-37.4	797 (69.3)		265 (46.1)		145 (25.2)		73 (6.4)		228 (19.8)
37.5-39.9	442 (65.6)		146 (43.3)		89 (26.4)		40 (5.9)		117 (17.4)
≥40.0	401 (77.1)		161 (61.9)		71 (27.3)		37 (7.1)		91 (17.5)
Overweight (n=51,796)									
0-2.4	1659 (67.5)		669 (54.4)		94 (7.7)		430 (17.5)		117 (4.8)
2.5-4.9	1546 (63.8)		607 (50.1)		81 (6.7)		370 (15.3)		125 (5.2)
5.0-7.4	2667 (59.9)		1022 (45.9)		177 (7.9)		637 (14.3)		257 (5.8)
7.5-9.9	4492 (55.6)		1686 (41.8)		301 (7.5)		1054 (13.1)		473 (5.9)
10.0-12.4	5819 (54.5)		2151 (40.3)		431 (8.1)		1211 (11.4)		712 (6.7)
12.5-14.9	7303 (54.7)		2709 (40.6)		621 (9.3)		1417 (10.6)		1014 (7.6)
15.0-17.4	7239 (50.5)		2419 (33.8)		758 (10.6)		1305 (9.1)		1338 (9.3)
17.5-19.9	6824 (51.1)		2240 (33.6)		740 (11.1)		1085 (8.1)		1459 (10.9)
20.0-22.4	5869 (53.3)		1873 (34.1)		792 (14.4)		815 (7.4)		1370 (12.5)
22.5-24.9	4471 (53.9)		1379 (33.2)		674 (16.3)		581 (7.0)		1132 (13.7)
25.0-27.4	3249 (57.0)		1026 (36.0)		493 (17.3)		364 (6.4)		889 (15.6)
27.5-29.9	2195 (60.1)		741 (40.6)		328 (18.0)		238 (6.5)		591 (16.2)
30.0-32.4	1483 (61.8)		454 (37.8)		249 (20.8)		156 (6.5)		433 (18.0)
32.5-34.9	918 (62.3)		282 (38.3)		179 (24.3)		82 (5.6)		291 (19.7)
35.0-37.4	628 (67.8)		215 (46.4)		116 (25.1)		51 (5.5)		195 (21.1)
37.5-39.9	368 (67.2)		115 (42.0)		72 (26.3)		33 (6.0)		123 (22.5)
≥40.0	385 (78.9)		145 (59.4)		83 (34.0)		40 (8.2)		98 (20.1)
Obese Class I (n=30,477)									
0-2.4	2085 (62.1)		813 (48.4)		128 (7.6)		452 (13.5)		206 (6.1)
2.5-4.9	1615 (60.4)		621 (46.5)		122 (9.1)		357 (13.4)		191 (7.2)
5.0-7.4	2502 (57.7)		929 (42.9)		222 (10.2)		474 (10.9)		310 (7.2)
7.5-9.9	3553 (56.8)		1277 (40.8)		348 (11.1)		697 (11.1)		519 (8.3)
10.0-12.4	4057 (55.2)		1408 (38.3)		447 (12.2)		743 (10.1)		682 (9.3)
12.5-14.9	4369 (56.1)		1523 (39.1)		508 (13.1)		654 (8.4)		869 (11.2)
15.0-17.4	4139 (54.3)		1342 (35.2)		528 (13.8)		591 (7.8)		964 (12.6)
17.5-19.9	3425 (55.6)		1099 (35.7)		474 (15.4)		437 (7.1)		867 (14.1)

© 2022 Lin D et al. *JAMA Network Open.*

20.0-22.4	2853 (57.5)		902 (36.4)		430 (17.4)		362 (7.3)		781 (15.8)
22.5-24.9	2106 (58.1)		635 (35.0)		347 (19.1)		258 (7.1)		567 (15.6)
25.0-27.4	1491 (60.3)		470 (38.0)		259 (21.0)		147 (6.0)		429 (17.4)
27.5-29.9	1050 (66.2)		339 (42.8)		184 (23.2)		100 (6.3)		282 (17.8)
30.0-32.4	680 (63.4)		216 (40.3)		115 (21.5)		53 (4.9)		195 (18.2)
32.5-34.9	474 (67.1)		149 (42.2)		85 (24.1)		52 (7.4)		150 (21.3)
35.0-37.4	294 (70.7)		96 (46.2)		55 (26.4)		28 (6.7)		88 (21.2)
37.5-39.9	179 (63.5)		53 (37.6)		37 (26.2)		22 (7.8)		49 (17.4)
≥40.0	227 (79.9)		88 (62.0)		40 (28.2)		15 (5.3)		48 (16.9)
Obese Class II (n=16,191)									
0-2.4	1829 (62.1)		708 (48.1)		158 (10.7)		327 (11.1)		265 (9.0)
2.5-4.9	1312 (59.9)		481 (43.9)		120 (11.0)		259 (11.8)		175 (8.0)
5.0-7.4	1672 (57.5)		601 (41.4)		194 (13.4)		277 (9.5)		282 (9.7)
7.5-9.9	2207 (56.8)		760 (39.1)		265 (13.6)		373 (9.6)		447 (11.5)
10.0-12.4	2223 (57.8)		737 (38.4)		297 (15.5)		367 (9.6)		450 (11.7)
12.5-14.9	2390 (60.5)		790 (40.0)		337 (17.1)		320 (8.1)		569 (14.4)
15.0-17.4	1959 (55.6)		611 (34.7)		301 (17.1)		259 (7.4)		500 (14.2)
17.5-19.9	1538 (58.2)		457 (34.6)		258 (19.5)		191 (7.2)		458 (17.3)
20.0-22.4	1232 (60.2)		377 (36.8)		221 (21.6)		125 (6.1)		375 (18.3)
22.5-24.9	934 (62.4)		299 (39.9)		152 (20.3)		95 (6.3)		282 (18.8)
25.0-27.4	651 (64.3)		197 (38.9)		103 (20.4)		80 (7.9)		209 (20.7)
27.5-29.9	450 (65.4)		140 (40.7)		86 (25.0)		53 (7.7)		124 (18.0)
30.0-32.4	326 (66.8)		105 (43.0)		59 (24.2)		28 (5.7)		104 (21.3)
32.5-34.9	193 (67.0)		60 (41.7)		41 (28.5)		17 (5.9)		69 (24.0)
≥35.0	319 (68.2)		119 (50.9)		59 (25.2)		30 (6.4)		99 (21.2)
Obese Class III (n=12,396)									
0-2.4	2182 (61.9)		790 (44.8)		262 (14.9)		371 (10.5)		331 (9.4)
2.5-4.9	1227 (59.9)		429 (41.9)		164 (16.0)		172 (8.4)		239 (11.7)
5.0-7.4	1458 (58.7)		494 (39.7)		188 (15.1)		241 (9.7)		315 (12.7)
7.5-9.9	1758 (58.0)		577 (38.1)		274 (18.1)		274 (9.0)		362 (12.0)
10.0-12.4	1704 (59.8)		545 (38.2)		286 (20.1)		230 (8.1)		430 (15.1)

© 2022 Lin D et al. *JAMA Network Open.*

12.5-14.9	1607 (59.9)		524 (39.1)		249 (18.6)		178 (6.6)		434 (16.2)
15.0-17.4	1316 (59.0)		373 (33.4)		238 (21.3)		144 (6.5)		398 (17.8)
17.5-19.9	1076 (62.5)		323 (37.5)		176 (20.4)		116 (6.7)		311 (18.1)
20.0-22.4	794 (63.1)		247 (39.3)		140 (22.3)		82 (6.5)		267 (21.2)
22.5-24.9	668 (66.8)		187 (37.4)		141 (28.2)		69 (6.9)		232 (23.2)
25.0-27.4	477 (71.4)		162 (48.5)		94 (28.1)		33 (4.9)		150 (22.5)
27.5-29.9	296 (67.6)		94 (42.9)		58 (26.5)		22 (5.0)		102 (23.3)
30.0-32.4	193 (67.0)		60 (41.7)		43 (29.9)		16 (5.6)		56 (19.4)
≥32.5	407 (72.4)		129 (45.9)		105 (37.4)		35 (6.2)		146 (26.0)
*, calculated as per neonate.									

© 2022 Lin D et al. *JAMA Network Open.*

eTable 6. Association of GWG Category and the Composite Outcome by Maternal BMI Category (Used as Main Study Sample)

Underweight (n= 5,333)				Normal-weight (n=84,617)				Overweight (n=51,796)		
Standardized GWG (kg)	aOR (95% CI)	*P*-value		Standardized GWG (kg)	aOR (95% CI)	*P*-value		Standardized GWG (kg)	aOR (95% CI)	*P*-value
				0-2.4	2.04 (1.71-2.42)	<0.001		0-2.4	1.71 (1.53-1.91)	<0.001
				2.5-4.9	2.20 (1.90-2.55)	<0.001		2.5-4.9	1.47 (1.31-1.64)	<0.001
< 7.5	3.00 (1.99-4.54)	<0.001		5.0-7.4	1.92 (1.75-2.12)	<0.001		5.0-7.4	1.23 (1.14-1.34)	<0.001
7.5-9.9	2.12 (1.59-2.81)	<0.001		7.5-9.9	1.62 (1.52-1.72)	<0.001		7.5-9.9	1.04 (0.98-1.10)	0.251
10.0-12.4	1.42 (1.17-1.74)	0.001		10.0-12.4	1.22 (1.17-1.28)	<0.001		10.0-12.4	0.99 (0.93-1.04)	0.582
12.5-14.9	1.33 (1.15-1.54)	<0.001		12.5-14.9	1.18 (1.13-1.22)	<0.001		12.5-14.9	0.99 (0.94-1.03)	0.555
15.0-17.4	0.89 (0.78-1.02)	0.088		15.0-17.4	0.81 (0.78-0.83)	<0.001		15.0-17.4	0.81 (0.78-0.85)	<0.001
17.5-19.9	0.79 (0.69-0.90)	0.001		17.5-19.9	0.79 (0.77-0.82)	<0.001		17.5-19.9	0.83 (0.79-0.87)	<0.001
20.0-22.4	0.81 (0.70-0.93)	0.004		20.0-22.4	0.85 (0.82-0.88)	<0.001		20.0-22.4	0.92 (0.87-0.97)	0.001
22.5-24.9	0.73 (0.62-0.87)	<0.001		22.5-24.9	0.82 (0.78-0.85)	<0.001		22.5-24.9	0.94 (0.89-1.00)	0.043
25.0-27.4	0.93 (0.74-1.17)	0.540		25.0-27.4	1.04 (0.99-1.10)	0.151		25.0-27.4	1.07 (0.99-1.14)	0.078
27.5-29.9	0.87 (0.65-1.16)	0.352		27.5-29.9	1.16 (1.08-1.24)	<0.001		27.5-29.9	1.21 (1.11-1.32)	<0.001
≥30.0	1.15 (0.89-1.49)	0.290		30.0-32.4	1.21 (1.11-1.32)	<0.001		30.0-32.4	1.28 (1.15-1.43)	<0.001
				32.5-34.9	1.28 (1.15-1.44)	<0.001		32.5-34.9	1.31 (1.14-1.51)	<0.001
				35.0-37.4	1.92 (1.63-2.26)	<0.001		35.0-37.4	1.68 (1.40-2.01)	<0.001
				37.5-39.9	1.57 (1.27-1.93)	<0.001		37.5-39.9	1.61 (1.27-2.03)	<0.001
				≥40.0	2.84 (2.17-3.71)	<0.001		≥40.0	2.98 (2.25-3.96)	<0.001
Obese Class I (n=30,477)				Obese Class II (n=16,191)				Obese Class III (n=12,396)		
Standardized GWG (kg)	aOR (95% CI)	*P*-value		Standardized GWG (kg)	aOR (95% CI)	*P*-value		Standardized GWG (kg)	aOR (95% CI)	*P*-value
0-2.4	1.23 (1.12-1.35)	<0.001		0-2.4	1.15 (1.04-1.28)	0.007		0-2.4	1.06 (0.96-1.17)	0.241
2.5-4.9	1.15 (1.03-1.27)	0.009		2.5-4.9	1.04 (0.92-1.16)	0.548		2.5-4.9	0.95 (0.85-1.08)	0.440
5.0-7.4	1.02 (0.94-1.11)	0.597		5.0-7.4	0.93 (0.84-1.03)	0.175		5.0-7.4	0.89 (0.80-1.00)	0.044
7.5-9.9	0.97 (0.91-1.04)	0.427		7.5-9.9	0.90 (0.82-0.98)	0.015		7.5-9.9	0.86 (0.78-0.96)	0.005
10.0-12.4	0.9 (0.85-0.96)	0.002		10.0-12.4	0.94 (0.86-1.03)	0.168		10.0-12.4	0.92 (0.83-1.03)	0.137
12.5-14.9	0.93 (0.87-0.99)	0.022		12.5-14.9	1.05 (0.96-1.15)	0.295		12.5-14.9	0.94 (0.85-1.05)	0.268

© 2022 Lin D et al. *JAMA Network Open.*

15.0-17.4	0.85 (0.80-0.91)	<0.001		15.0-17.4	0.83 (0.76-0.91)	<0.001		15.0-17.4	0.90 (0.80-1.01)	0.065
17.5-19.9	0.91 (0.85-0.98)	0.009		17.5-19.9	0.94 (0.85-1.04)	0.246		17.5-19.9	1.05 (0.92-1.20)	0.471
20.0-22.4	0.99 (0.92-1.07)	0.878		20.0-22.4	1.02 (0.90-1.15)	0.781		20.0-22.4	1.08 (0.93-1.26)	0.303
22.5-24.9	1.01 (0.93-1.11)	0.793		22.5-24.9	1.11 (0.97-1.28)	0.140		22.5-24.9	1.28 (1.07-1.52)	0.006
25.0-27.4	1.11 (0.99-1.23)	0.063		25.0-27.4	1.21 (1.02-1.43)	0.030		25.0-27.4	1.61 (1.29-2.01)	<0.001
27.5-29.9	1.43 (1.25-1.64)	<0.001		27.5-29.9	1.27 (1.03-1.56)	0.025		27.5-29.9	1.32 (1.01-1.72)	0.038
30.0-32.4	1.25 (1.07-1.48)	0.007		30.0-32.4	1.35 (1.06-1.73)	0.017		30.0-32.4	1.27 (0.92-1.75)	0.152
32.5-34.9	1.48 (1.21-1.82)	<0.001		32.5-34.9	1.36 (0.99-1.88)	0.060		≥32.5	1.67 (1.31-2.13)	<0.001
35.0-37.4	1.79 (1.36-2.36)	<0.001		≥35.0	1.43 (1.11-1.85)	0.006				
37.5-39.9	1.28 (0.93-1.75)	0.133								
≥40.0	2.87 (1.97-4.20)	<0.001								
aORs were obtained from multivariable models adjusted for maternal age, race, nulliparity, mode of conception, smoking status, and neonatal sex combination.										

© 2022 Lin D et al. *JAMA Network Open.*

eTable 7. Comparison Between IOM 2009 and Current Optimal GWG Ranges and GWG Corresponding to Maternal Fetal Corpulence Symbiosis (MFCS)

Maternal prepregnancy BMI	IOM 2009 for singletons	PROPOSED LINEAR MODEL GWG to have AGA newborns Robillard et al. 2018 (OptGWG=-1.2ppBMI+42)		IOM 2009 for twin pregnancies (Standardized for 36 weeks)	Optimal GWG ranges for twin pregnancies based on statistical-based approach	Optimal GWG ranges for twin pregnancies based on outcome-based approach	PROPOSED LINEAR MODEL Standardized GWG to have AGA newborns (GWG (kg)=-0.932ppBMI (kg/m^2) +41.5)	
Underweight women < 18.5kg/m²	12.5-18 kg	22.8 kg (16 kg/m^2)<br>21.6 kg (17 kg/m^2)<br>20.4 kg (18 kg/m^2)	20 to 23 kg	-	15.9 to 22.7 kg	17.5 to 24.9 kg	26.6 kg (16 kg/m^2)<br>25.7 kg (17 kg/m^2)<br>24.7 kg (18 kg/m^2)	24 to 27 kg
Normal weight women 18.5-24.9 kg/m²	11.5-16 kg	19.2 kg (19 kg/m^2)<br>18 kg (20 kg/m^2)<br>16.8 kg (21 kg/m^2)<br>15.6 kg (22 kg/m^2)<br>14.4 kg (23 kg/m^2)<br>13.2 kg (24 kg/m^2)	13 to 19 kg	16.3 to 23.8 kg	15.4 to 22.7 kg	15.0 to 24.9 kg	23.8 kg (19 kg/m^2)<br>22.9 kg (20 kg/m^2)<br>21.9 kg (21 kg/m^2)<br>21 kg (22 kg/m^2)<br>20.1 kg (23 kg/m^2)<br>19.1 kg (24 kg/m^2)	19 to 24 kg
Overweight women 25-29.9 kg/m²	7-11.5 kg	12 kg (25 kg/m^2)<br>10.8 kg (26 kg/m^2)<br>9.6 kg (27 kg/m^2)<br>8.4 kg (28 kg/m^2)<br>7.2 kg (29 kg/m^2)	7 to 12 kg	13.7 to 22.1 kg	12.7 to 22.2 kg	15.0 to 24.9 kg	18.2 kg (25 kg/m^2)<br>17.3 kg (26 kg/m^2)<br>16.3 kg (27 kg/m^2)<br>15.4 kg (28 kg/m^2)<br>14.5 kg (29 kg/m^2)	14 to 18 kg
Obesity class I 30-34.9 kg/m²	5-9 kg	6 kg (30 kg/m^2)<br>4.8 kg (31 kg/m^2)<br>3.6 kg (32 kg/m^2)<br>2.4 kg (33 kg/m^2)<br>1.2 kg (34 kg/m^2)	1 to 6 kg	11 to 18.6 kg	10.0 to 20.0 kg	10.0 to 19.9 kg	13.5 kg (30 kg/m^2)<br>12.6 kg (31 kg/m^2)<br>11.7 kg (32 kg/m^2)<br>10.7 kg (33 kg/m^2)<br>9.8 kg (34 kg/m^2)	10 to 14 kg
Obesity class II 35-39.9 kg/m²	5-9 kg	0 kg (35 kg/m^2)<br>-1.2 kg (36 kg/m^2)<br>-2.4 kg (37 kg/m^2)<br>-3.6 kg (38 kg/m^2)<br>-4.8 kg (39 kg/m^2)	0 to - 5kg	11 to 18.6 kg	7.7 to 18.1 kg	7.5 to 17.4 kg	8.9 kg (35 kg/m^2)<br>7.9 kg (36 kg/m^2)<br>7 kg (37 kg/m^2)<br>6.1 kg (38 kg/m^2)<br>5.2 kg (39 kg/m^2)	5 to 9 kg

© 2022 Lin D et al. *JAMA Network Open.*

Obesity class III<br>Over 40 kg/m^2	5-9 kg	-6 kg (40 kg/m^2)<br>-7.2 kg (41 kg/m^2)<br>-8.4 kg (42 kg/m^2)<br>-9.6 kg (43 kg/m^2)<br>-10.8 kg (44 kg/m^2)<br>-12 kg (45 kg/m^2)<br>-13.2 kg (46 kg/m^2)	-6 kg	11 to 18.6 kg	5.9 to 16.3 kg	5.0 to 9.9 kg	4.2 kg (40 kg/m^2)<br>3.3 kg (41 kg/m^2)<br>2.4 kg (42 kg/m^2)<br>1.4 kg (43 kg/m^2)<br>0.5 kg (44 kg/m^2)<br>-0.4 kg (45 kg/m^2)<br>-1.4 kg (46 kg/m^2)	-1 to 4 kg

© 2022 Lin D et al. *JAMA Network Open.*

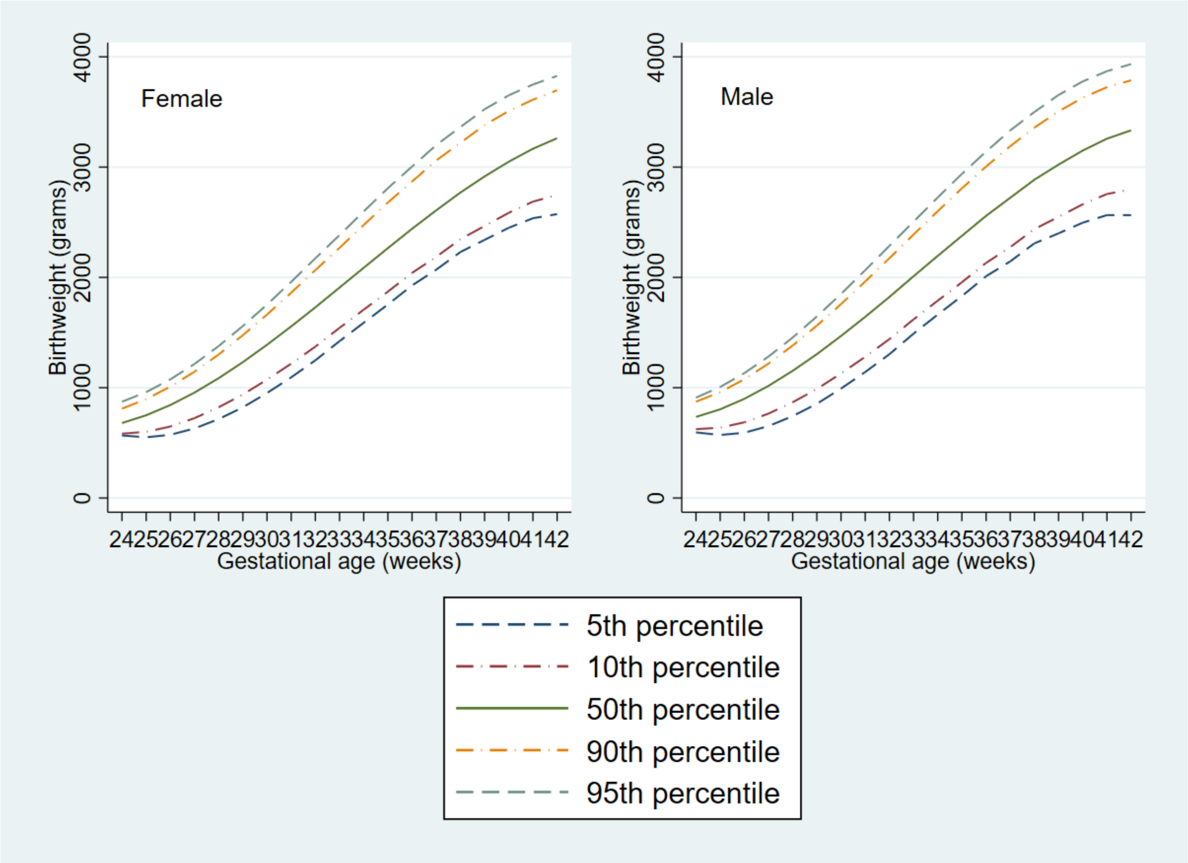

eFigure 1. Sex-Specific Birthweight Percentile Curves in Twins (Used as Both Main Study Sample and Validation Sample)

© 2022 Lin D et al. *JAMA Network Open.*

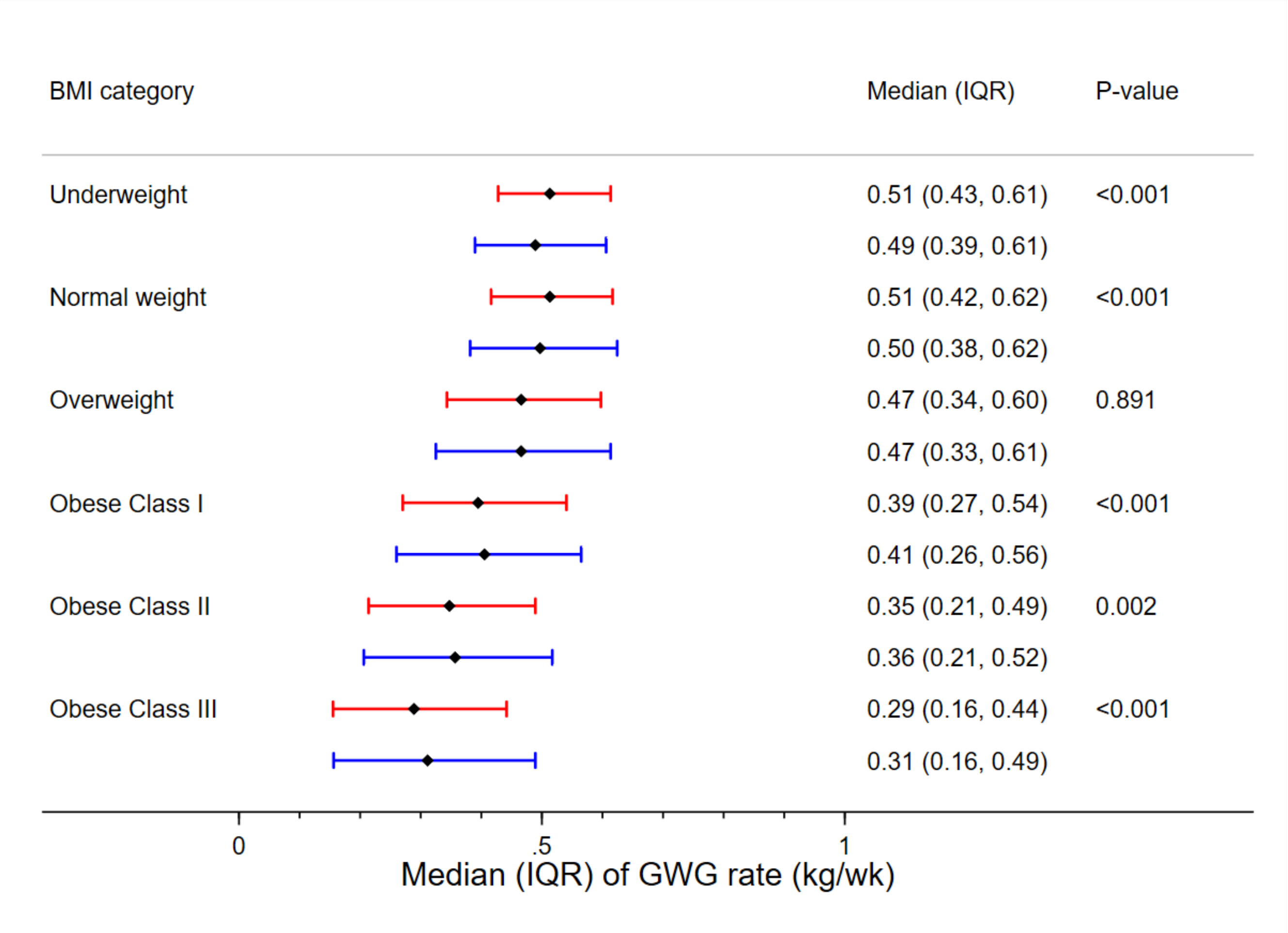

eFigure 2. The Median and Interquartile Range (IQR) of GWG Rate in Low-Risk and High-Risk Subgroups Stratified by BMI Category (Used as Main Study Sample). Red line, low-risk subgroup; blue line, high-risk subgroup.

© 2022 Lin D et al. *JAMA Network Open.*

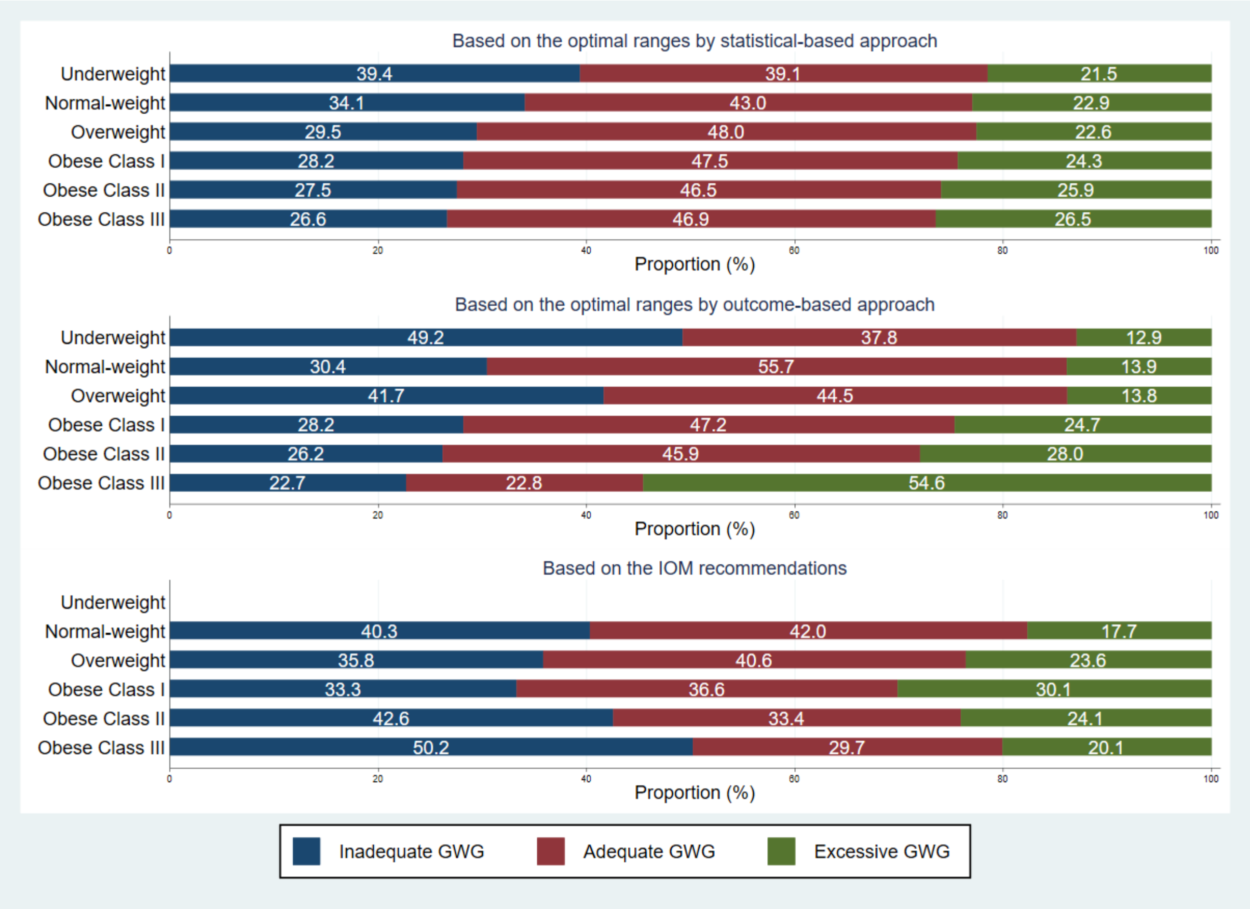

eFigure 3. Proportions of Inadequate, Adequate and Excessive GWG Defined Based on the Optimal Ranges (Statistical-Based and Outcome-Based Approach) and the IOM Recommendations by Prepregnancy BMI Category (Used as Validation Sample). *, the IOM recommendations are not available for underweight women with twin pregnancies.

© 2022 Lin D et al. *JAMA Network Open.*

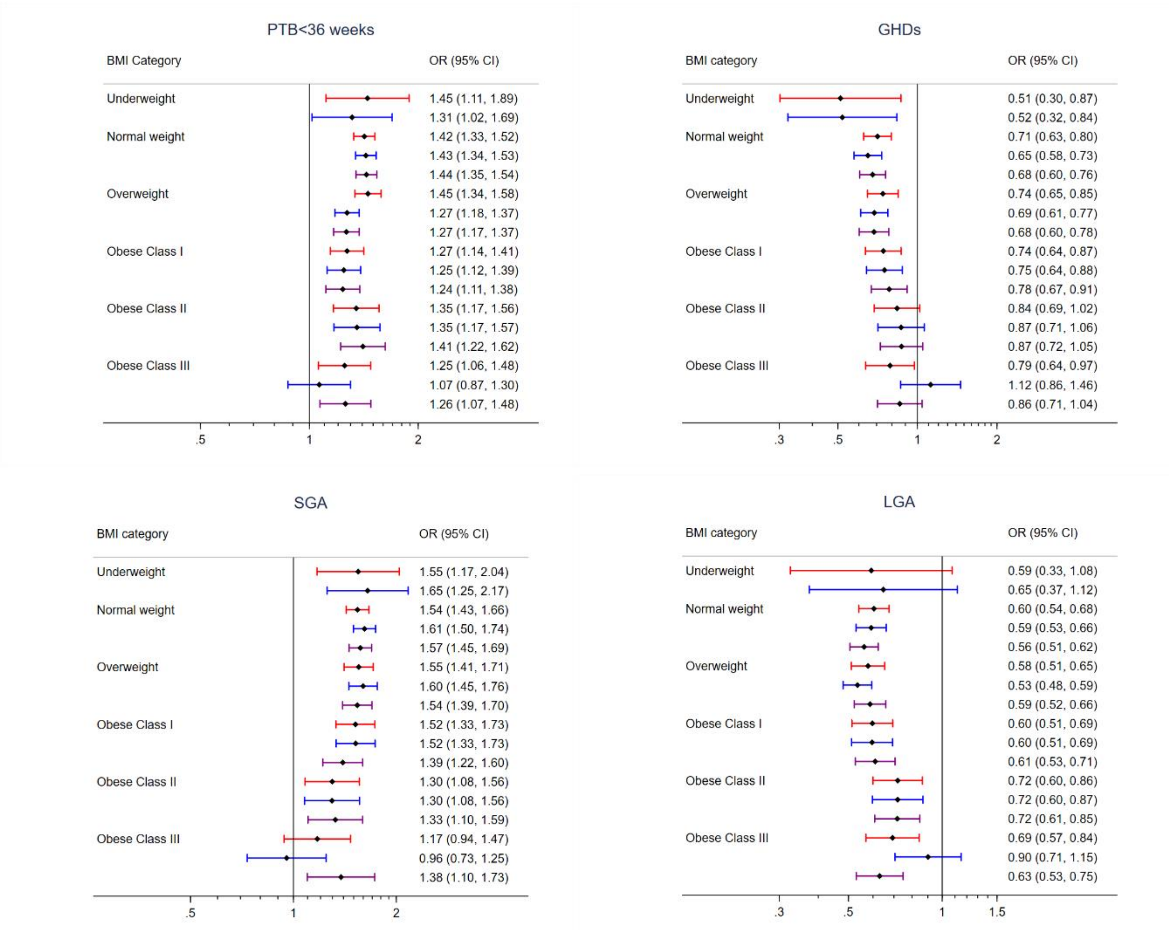

eFigure 4. Adjusted Odd Ratios (aORs) for Perinatal Outcomes of Inadequate GWG (Used as Validation Sample). Red line: based on the optimal ranges by statistical-based approach; Blue line: based on the optimal ranges by outcome-based approach; Purple line: based on the IOM recommendations.

© 2022 Lin D et al. *JAMA Network Open.*

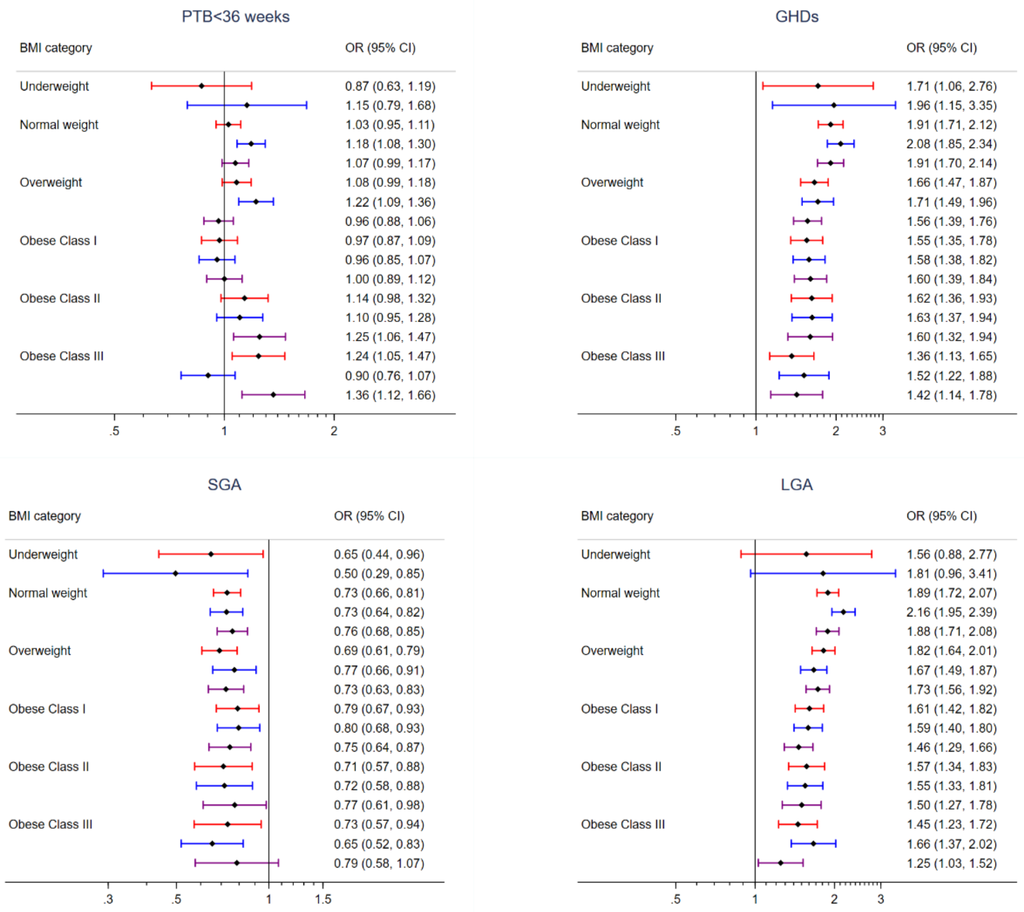

eFigure 5. Adjusted Odd Ratios (aORs) for Perinatal Outcomes of Excessive GWG (Used as Validation Sample). Red line: based on the optimal ranges by statistical-based approach; Blue line: based on the optimal ranges by outcome-based approach; Purple line: based on the IOM recommendations.

© 2022 Lin D et al. *JAMA Network Open.*
